# Live microbials to boost Anti-SARS-CoV-2 immunity clinical trial (Live BASIC trial): a triple-blind randomized controlled trial

**DOI:** 10.1007/s15010-025-02697-4

**Published:** 2025-11-26

**Authors:** Daniel B. Horton, Rahul Ukey, Abhilasha Madhvi, Tracy Andrews, Veenat Parmar, Nancy Reilly, Sanna M. Mäkelä, Jonathan Peterson, Leah Hustad, Gloriana Wong, Emily S. Barrett, Natalie Bruiners, Jeffrey L. Carson, Kylie Getz, Patricia Greenberg, Alicia Iizuka, Jason Roy, Alexander W. Pastuszak, Markus J. Lehtinen, Martin J. Blaser, Reynold A. Panettieri, Maria Laura Gennaro

**Affiliations:** 1 Department of Pediatrics, Rutgers Robert Wood Johnson Medical School, New Brunswick, NJ USA; 2 Rutgers Center for Pharmacoepidemiology and Treatment Science, Institute for Health, Health Care Policy and Aging Research, 112 Paterson Street, New Brunswick, NJ USA; 3 Department of Biostatistics and Epidemiology, Rutgers School of Public Health, Piscataway, NJ USA; 4 Public Health Research Institute, Department of Medicine, New Jersey Medical School, Rutgers University, Newark, NJ USA; 5 Center for Advanced Biotechnology and Medicine, Rutgers University, Piscataway, NJ USA; 6 Rutgers Institute for Translational Medicine & Science, New Brunswick, NJ USA; 7 IFF Health & Biosciences, Kantvik, Finland; 8 IFF Health & Biosciences, Wilmington, DE USA; 9 CME Clinical Consulting, Fargo, ND USA; 10 Vesta Healthcare, New York, NY USA; 11 Environmental and Occupational Health Sciences Institute, Rutgers University, Piscataway, NJ USA; 12 Department of Medicine, Rutgers Robert Wood Johnson Medical School, New Brunswick, NJ USA; 13 Division of Urology, Department of Surgery, University of Utah School of Medicine, Salt Lake City, UT USA; 14 Department of Medicine, Rutgers New Jersey Medical School, Newark, NJ USA

**Keywords:** Probiotics, Randomized controlled trial, COVID-19, Secondary prevention

## Abstract

**Purpose:**

With waning immunity and vaccine hesitancy, the COVID-19 pandemic continues to pose risks. A live microbial consortium (OL-1) with bacteria containing potentially cross-reactive antigens (CRAGs) stimulates anti-SARS-CoV-2 immune responses in vitro/vivo. We evaluated OL-1’s efficacy in enhancing anti-SARS-CoV-2 immunity in unvaccinated, previously infected adults.

**Methods:**

We conducted a pilot, parallel-group, triple-blind randomized controlled trial in 2021–2022 involving 52 generally healthy adults ages 18–60, unvaccinated against COVID-19, with SARS-CoV-2 infection ≥ 4 months prior. Participants received 21 days of either standard-dose OL-1, high-dose OL-1, or placebo. The primary outcome was change in plasma anti-SARS-CoV-2 IgG titers from baseline to Day 21. Secondary efficacy outcomes included changes through Day 42, interferon gamma (IFNg) release from stimulated peripheral blood mononuclear cells, and new SARS-CoV-2 infections. Safety was assessed through adverse events.

**Results:**

Significant increases in plasma IgG levels were observed by Day 42 in the standard-dose OL-1 group (*n* = 17) compared to placebo (*n* = 18) (*p* = 0.02). No significant changes were observed in the high-dose group (*n* = 17). Marginal increases in IFNg release were observed in standard-dose recipients after stimulation with CD4+-specific CRAG and SARS-CoV-2 peptides and TLR7 ligands; only changes post-TLR7 ligand stimulation were significant. No new SARS-CoV-2 infections were detected. The most common adverse events overall were mild gastrointestinal symptoms; headaches were more frequent in OL-1 recipients.

**Conclusion:**

The live microbial consortium OL-1 was well-tolerated and associated with slightly increased anti-SARS-CoV-2 IgG levels in previously infected, unvaccinated adults at standard, but not high, dosage. Further research should confirm these findings and their clinical implications in larger populations.

This study was registered on ClinicalTrials.gov (NCT04847349) on April 14, 2021.

**Supplementary Information:**

The online version contains supplementary material available at 10.1007/s15010-025-02697-4.

## Introduction

In the wake of the COVID-19 pandemic, COVID-19 remains an important cause of ongoing morbidity, mortality, and healthcare utilizationf [1]. SARS-CoV-2 infections produce detectable immune responses in most infected individuals [2], but anti-SARS-CoV-2 immunity wanes over time, and re-infections are common [3–5]. Approved vaccines against SARS-CoV-2 are widely available, effective, and safe, although vaccine-induced immunity also decreases over time [6, 7]. Rates of anti-SARS-CoV-2 vaccination vary widely across different regions of the world and have been declining over time despite the emergence of new SARS-CoV-2 variants, new waves of infection, and the development of prolonged illnesses (long Covid) in some infected persons [1, 8, 9]. In the face of ongoing risks of (re)infection, incomplete individual and population-level immunity, and rising vaccine hesitancy [10–12], other means of protection against COVID-19 are desirable.

Probiotic bacterial strains have been investigated as potential strategies for the treatment and prevention of infectious diseases due to mechanisms including competition against pathogens, reinforcement of the gut epithelial barrier, and modulation of host immunity [13]. During the COVID-19 pandemic, probiotics were studied as adjunctive treatments for COVID-19 in various care settings, with mixed results [14]. In one randomized controlled trial (RCT) of SARS-CoV-2-infected adults not requiring hospitalization (*n* = 300), participants assigned to use of a live microbial consortium were nearly twice as likely to be symptom-free and without detectable virus 30 days after randomization (53% vs. 28%) and had greater reduction of symptoms, nasopharyngeal viral load, and lung infiltrates [15]. Modification of microbiota has also been explored as an alternate approach to enhancing immunity against SARS-CoV-2. In one RCT examining the use of probiotics as postexposure prophylaxis in people with recent household exposure to COVID-19 (*n* = 182), participants randomized to receive *Lacticaseibacillus rhamnosus* GG had fewer symptoms and longer times to COVID-19 diagnosis than the placebo group, although the lower incidence of COVID-19 in the probiotic group was not statistically significant (8.8% vs. 15.4%, *p* = 0.17) [16]. 

We previously showed that live bacterial (probiotic) consortia could catalyze an immune response against SARS-CoV-2 in vitro and in vivo [17]. In a ferret model of SARS-CoV-2 infection, two distinct live microbial consortia (named OL-1 and OL-2) both significantly reduced the intranasal viral load and enhanced expression of type I interferon (IFN) and chemokines in lung tissue. In human peripheral blood monocyte-derived macrophages and dendritic cell, both consortia induced IL-12 and IFN gamma (IFNg) production as well as transcriptomic pathways associated with antiviral responses. In further preliminary analyses, five live bacterial strains in one consortium (OL-1, see **Investigational Product**) were identified as having potential cross-reactive antigens (CRAGs) to SARS-CoV-2 constituents that could stimulate anti-SARS-CoV-2 immune responses [18]. Supporting the hypothesis of cross-reactivity, polyclonal anti-SARS-CoV-2 spike antibodies bound to multiple proteins in these live bacteria, and antibody binding was blocked by introducing purified spike antigens to the assay (unpublished data). CRAGs can theoretically serve several functions: (1) priming the immune system to mount a faster, more effective immune response against primary infection (similar to vaccination); (2) boosting the immune response from vaccination; and (3) enhancing and extending immunity among those previously infected with SARS-CoV-2. We examined the third potential function of the selected live bacteria: boosting immunity. The safety of the live bacteria under this study has been well-established in pre-clinical and clinical studies for other indications [19–23]. 

As such, we conducted a pilot RCT examining the preliminary efficacy of OL-1 given to boost antiviral immunity of unvaccinated, previously SARS-CoV-2-infected persons.

## Materials & methods

### Trial design

The Live Microbials to Boost Anti-SARS-CoV-2 Immunity Clinical Trial (Live BASIC) Trial was designed as a pilot, parallel-group, triple-blind randomized controlled trial of two doses (standard and high dose) of the live microbial consortium OL-1 versus placebo, taken daily for 21 days (**Supplementary Methods**, Supplementary Fig. 1). Participants were randomized 1:1:1 to treatment groups using permuted block randomization. The study funder provided the randomization tables, to which only the research pharmacist had access throughout the study. All participants and other study investigators and staff with direct contact with study participants or their data were blinded to treatment assignments (**Supplementary Methods**). Potential participants were screened for eligibility with an online form, and no study procedures were conducted until participants provided informed consent. The eligibility criteria (**Participants**) changed during the trial to allow participants with a documented illness consistent with COVID-19 and positive SARS-CoV-2 antibody testing to participate.

### Participants

Participants were generally healthy English-speaking adults ages 18–60 with documented SARS-CoV-2 infection occurring at least 4 months prior to enrollment (see **Supplementary Methods** for full eligibility criteria). Individuals were excluded if they were already (or soon to become) vaccinated against COVID-19, had COVID-19 within the prior 4 months, had regularly used probiotic supplements in the last 3 months, had a history of severe asthma, immunodeficiency, inflammatory bowel disease, or irritable bowel syndrome, or were pregnant or breastfeeding. Recruitment initially began in New Jersey, New York, and Pennsylvania, but later expanded to Georgia, Minnesota, and Texas. Participants were recruited from databases of people who tested positive for SARS-CoV-2 through Vault Health or other ongoing studies at Rutgers University [24, 25]. Vault Health provides remote and at-home healthcare to patients across the United States as well as clinical research services to enable decentralized clinical studies, including maintenance of a secure virtual data platform, which was used for participant screening and data collection. Participants were also recruited via advertisements on social media (Facebook, Twitter, Reddit) and through the study website maintained by Vault Health.

### Interventions

Participants were instructed to take the assigned investigational product daily with breakfast for 21 days starting after the baseline evaluation (**Data Collection**). OL-1 and placebo were formulated as indistinguishable capsules. The OL-1 consortium contained a mixture of 5 live bacterial strains: *Bifidobacterium animalis* subsp. *lactis* (Bl-04), *Bifidobacterium longum* subsp. *infantis* (Bi-26™), *Lacticaseibacillus rhamnosus* (Lr-32^®^), *Lacticaseibacillus paracasei* (Lpc-37), *Ligilactobacillus salivarius* (Ls-33^®^). The standard-dose product consisted of at least 43 × 10^9^ colony forming units (CFU) in total, while the high-dose product consisted of at least 75 × 10^9^ CFU in total (**Supplementary Methods**). Both doses of live microbials used in OL-1 were comparable to those used previously in other human clinical trials of the individual microbial strains and corresponding commercial products [20, 26–29]. 

### Data collection

The Live BASIC Trial was fully decentralized, with shipment of kits with the investigational products directly to participants homes; conduct of study visits (baseline, Day 21, Day 42) and collection of biospecimens (blood, nasal wash, and saliva) by trained study personnel in participants’ homes or, when preferred, nearby clinical sites; and collection of all other data virtually via online questionnaires on Vault Health’s secure virtual data platform or secure video interactions with study staff (Supplementary Table 1). Questionnaire data included baseline demographics, medical history including details about prior SARS-CoV-2 infection, medication and supplement use, smoking history, and baseline symptoms related to prior SARS-CoV-2 infection. Weekly follow-up questionnaires assessed symptoms from prior or new SARS-CoV-2 infection (e.g., fever, fatigue, loss of smell or taste, headache), adverse events (AEs), adherence to the investigational product (through Day 21), and other interval events (e.g., vaccination against COVID-19). Additional information related to events or potential serious AEs was obtained as needed via virtual interactions with study staff. Adherence was confirmed by counting any remaining capsules in kits shipped back to the research pharmacist after the treatment period.

### Outcomes

The pre-specified primary trial outcome was the change in plasma anti-SARS-CoV-2 IgG titer between baseline and Day 21, selected as a proxy for clinical response in this pilot study (see **Supplementary Methods**). Plasma anti-SARS-CoV-2 IgG titer was determined by enzyme-linked immunosorbent assay (ELISA) targeting the Receptor Binding Domain (RBD) of the S1 subunit of the viral spike protein, a primary target of neutralizing antibodies [30, 31]. Secondary immunologic efficacy outcomes included changes between baseline and Day 21 and/or 42 of: plasma anti-SARS-CoV-2 IgG titer (Day 42); plasma anti-SARS-CoV-2 IgA antibody; nasal anti-SARS-CoV-2 IgA antibody; cytokine release (IFNg) in response to stimulation of peripheral blood mononuclear cells (PBMCs) with SARS-CoV-2-derived peptides; and plasma antibodies binding probiotic bacterial antigens (post-hoc) (**Supplementary Methods**). Secondary clinical efficacy outcomes included: change in any baseline long-term symptoms or sequelae of prior SARS-CoV-2 infection, and incidence of new (repeat) SARS-CoV-2 infections, as assessed in saliva specimens. Secondary safety outcomes include AEs and serious AEs.

### Statistical analysis

A total of 54 eligible participants were targeted for enrollment (18 per study arm) to achieve at least 15 evaluable subjects per arm based on estimated 15% dropout. This sample size was based on having 0.78–0.85 power for a hypothetical mean change of 1.1–1.2 in log titer of plasma anti-SARS-CoV-2 IgG between baseline and Day 21 in each active treatment group relative to placebo. Participants who received COVID-19 vaccination during the study period were removed from the study and excluded from further evaluation.

Initial analyses of the primary outcome were conducted based on pairwise group comparisons (A vs. B, A vs. C, and B vs. C), without knowledge of which group corresponded to which assigned treatment. Following these analyses, arm-specific treatment assignments were fully unblinded to complete all analyses (**Supplementary Methods**).

Baseline characteristics were summarized using medians and interquartile range (IQR) or frequencies and percentages, stratified by assigned group. Between-group differences in the change in log-transformed anti-SARS-CoV-2 IgG titers from baseline to Days 21 and 42 were estimated using repeated measures linear regression with a random effect for time and represented by marginal means with 95% confidence intervals (CIs) to contrast values between each active treatment groups and placebo across visits (**Supplementary Methods**). Non-transformed IFNg release and probiotic antibody data were analyzed similarly. In secondary models, analyses were conducted adjusting for age. For binary outcomes, such as adverse events or new infections, chi-square or Fisher’s exact tests were used to compare the assigned groups.

**Fig. 1:**
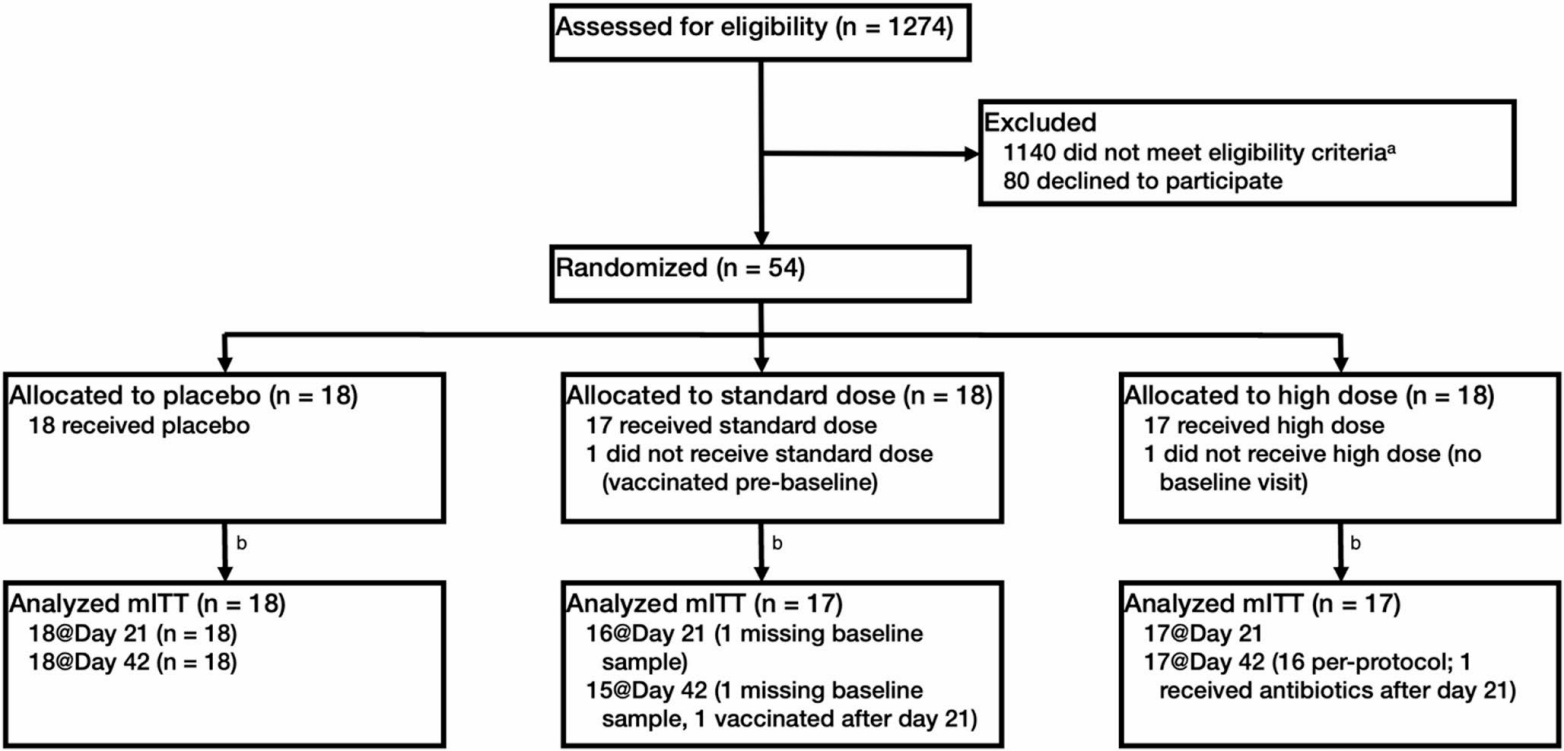
Live BASIC Trial CONSORT Diagram. mITT, modified intention-to-treat population. ^a^ Reasons for ineligibility: Prior vaccination (335), Regular probiotic use (235), Ineligible chronic conditions (233), Recent COVID-19 reinfection or symptoms (156), Recent hospitalization (not for COVID-19) (37), Antibiotics in last 2 weeks (32), Excessive drug use (25), Recent immunosuppression (22), Non-English speaker (11), Intent to receive vaccine soon (9), Pregnant or breastfeeding (9), Insufficient documentation of infection (8), Other reasons (28). ^b^ No participants were lost to follow-up or discontinued the intervention

Primary analyses were conducted following intention-to-treat principles. Analyses were only performed on participants who had study visits (modified intention-to-treat population, mITT) since blood samples used to assess the primary outcome were available from those visits (Fig. 1). Secondary per-protocol analyses excluded subjects who received antibiotics during the study period, received a COVID-19 vaccination during the study period, tested positive for SARS-CoV-2 during the study period, or consumed fewer than 14 capsules of the investigational product.

Analyses were completed using SAS 9.4, R 4.0.3, and Stata 17.0. *P*-values less than 0.05 were considered statistically significant, and no adjustments for multiple comparisons were made.

## Results

### Participant characteristics

A total of 54 participants were enrolled between April and November 2021, and follow-up continued through January 2022. Two enrolled participants lacked baseline and follow-up data and did not receive any investigational product (one each due to: vaccination before the baseline study visit; scheduling issues preventing evaluation). Thus, the final mITT study population consisted of 52 randomized subjects who began taking the investigational product (Fig. 1; Table 1). By chance, the randomized groups differed in age, with the standard-dose group being significantly younger (median age 37 years [interquartile range (IQR) 34–43]) than the placebo group (median 50 years [IQR 38–53]) or high-dose group (median 54 years [IQR 47–57]). No other significant differences were noted across treatment groups. Most participants were White, non-Hispanic, non-smokers, and had no chronic disease. Approximately half of participants took some medication (frequently dietary supplements) at baseline. Over half of participants had SARS-CoV-2 infections between 4 and 8 months prior to baseline, and most participants reported having had either moderate, mild, or no symptoms. About one in five participants (10, 19%) reported having complications from COVID-19, most commonly post-acute sequelae of COVID-19 (PASC) (Table 1). One participant received COVID-19 vaccine after Day 21 and was withdrawn early (Fig. 1). Adherence to the study product was high and did not differ by treatment arm (Supplementary Table 2).

**Table 1:** Baseline characteristics of study participants

Type	Characteristic	Placebo (*n* = 18)	Standard-Dose (*n* = 17)	High-Dose (*n* = 17)	*p*-value
Demographics	Age				< 0.001
	18–40	5 (28%)	12 (71%)	1 (6%)	
	41–60	13 (72%)	5 (29%)	16 (94%)	
	Female gender	12 (67%)	13 (76%)	10 (59%)	0.58
	Race				0.66
	White	16 (89%)	14 (82%)	17 (100%)	
	Black	0 (0%)	1 (6%)	0 (0%)	
	Declined	1 (6%)	1 (6%)	0 (0%)	
	Other	1 (6%)	1 (6%)	0 (0%)	
	Non-Hispanic Ethnicity	16 (89%)	12 (71%)	15 (88%)	0.30
	NJ/NY residence	10 (56%)	10 (59%)	11 (65%)	0.94
Baseline health	Temperature, median (IQR)	97.6 (97.6, 98.1)	97.6 (97.4, 97.9)	97.8 (97.4, 98.0)	0.60
	Pulse, median (IQR)	72 (58–87)	83 (73–84)	72 (68–80)	0.15
	SBP, median (IQR)	124 (108–132)	112 (105–126)	118 (115–130)	0.48
	DBP, median (IQR)	71 (63–81)	78 (68–80)	75 (66–83)	0.74
	BMI				0.27
	Normal weight (< 25)	9 (50%)	5 (29%)	10 (59%)	
	Overweight (25-29.9)	4 (22%)	8 (47%)	6 (35%)	
	Obese (30-39.9)	4 (22%)	4 (24%)	1 (6%)	
	Smoking				0.61
	Never	11 (61%)	12 (71%)	14 (82%)	
	Prior	5 (28%)	3 (18%)	3 (18%)	
	Current	2 (11%)	2 (12%)	0 (0%)	
	Baseline chronic disease^a^	4 (22%)	2 (12%)	4 (24%)	0.74
	Baseline medication use	10 (56%)	9 (53%)	6 (35%)	0.48
	Prior receipt of probiotics^b^	1 (6%)	2 (12%)	2 (12%)	0.73
	Pain at baseline				0.62
	None	14 (78%)	13 (76%)	14 (82%)	
	Mild	4 (22%)	2 (12%)	2 (12%)	
	Moderate	0 (0%)	2 (12%)	1 (6%)	
	Anxiety or depression at baseline				0.39
	None	15 (83%)	11 (65%)	16 (94%)	
	Mild	2 (11%)	3 (18%)	1 (6%)	
	Moderate	1 (6%)	2 (12%)	0 (0%)	
	Severe	0 (0%)	1 (6%)	0 (0%)	
	Health scale at baseline, median (IQR)^c^	90 (85–94)	90 (80–95)	90 (85–92)	0.97
	Baseline symptoms (any)				0.15
	None	0 (0%)	0 (0%)	1 (6%)	
	Mild	2 (11%)	4 (24%)	7 (41%)	
	Moderate	12 (67%)	8 (47%)	4 (24%)	
	Severe	4 (22%)	3 (18%)	4 (24%)	
	Very Severe	0 (0%)	2 (12%)	1 (6%)	
COVID-19	Type of positive COVID-19 test				0.55
	PCR	15 (83%)	11 (65%)	14 (82%)	
	Antigen	1 (6%)	4 (24%)	1 (6%)	
	Antibody	2 (11%)	2 (12%)	2 (12%)	
	First positive COVID-19 test to baseline				0.85
	4 to < 8 months	9 (50%)	8 (47%)	11 (65%)	
	8 to < 12 months	7 (39%)	6 (35%)	4 (24%)	
	≥12 months	2 (11%)	3 (18%)	2 (12%)	
	Complications from COVID-19	5 (28%)	3 (18%)	2 (12%)	0.56
	PASC	4 (22%)	3 (18%)	0 (0%)	0.17
	COVID-19 severity				0.10
	Mild or no symptoms	2 (11%)	4 (24%)	8 (47%)	
	Moderate symptoms	12 (67%)	8 (47%)	4 (24%)	
	Severe symptoms	4 (22%)	5 (29%)	5 (29%)	

BMI body mass index; DBP diastolic blood pressure; HR heart rate; IQR interquartile range; PASC, post-acute sequelae of COVID-19; PCR polymerase chain reaction; SBP systolic blood pressure; SD standard deviation.

^a^ Per protocol, participants must be in general good health, allowing for pre-existing chronic disease that are well-controlled but excluding immunodeficiencies, chronic gut disorders, severe respiratory disease, and use of immunosuppressive medications.

^b^ Any probiotics in the prior 3 months; subjects with regular use of probiotics (e.g., more than 3 days per week) were excluded.

^c^ Self assessment of present health on a scale from 0 (worst health imaginable) to 100 (best health imaginable)

### Efficacy

#### Primary outcome

**Fig. 2:**
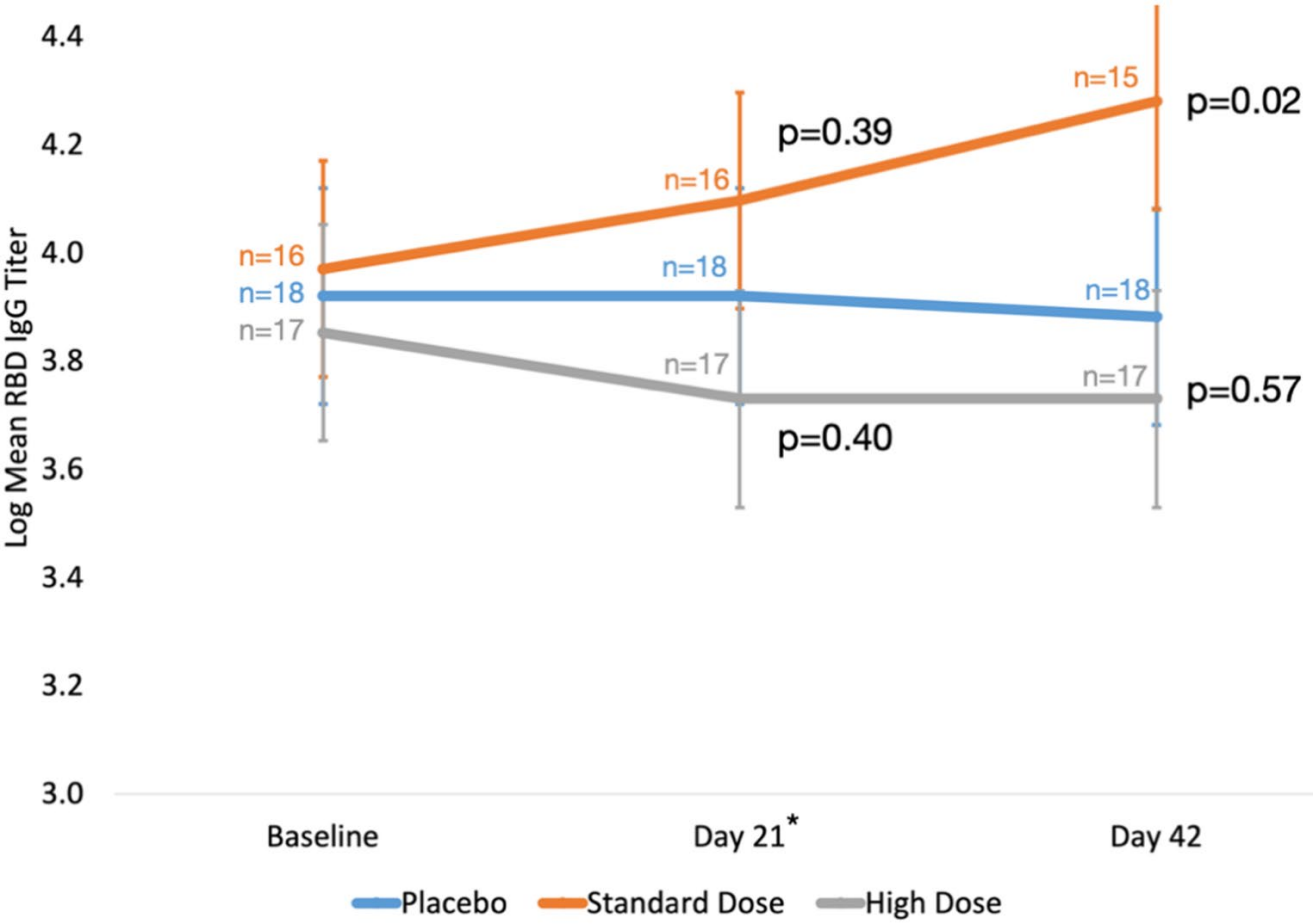
Change in log-transformed plasma RBD IgG titer (primary outcome). IgG, immunoglobulin G; RBD, Receptor Binding Domain of the S1 subunit of the viral spike protein. *Primary outcome assessment at Day 21. Bars indicate 95% confidence intervals

There were no significant differences in plasma IgG titers between groups at the primary endpoint assessment at Day 21. However, mean plasma IgG levels in the standard-dose group rose over Days 21 and 42 relative to placebo, with a significant difference of 0.35 log titer (95% CI 0.06–0.64) (*p* = 0.02) by Day 42. This difference corresponded to 1.9-fold relative increase in mean IgG titers. No significant changes in mean plasma IgG levels were observed in the high-dose group or combined treatment groups relative to placebo (Fig. 2; Table 2). These results were unchanged after adjustment for age (Table 2).

**Table 2:** Change in log-transformed plasma RBD IgG titer (primary outcome)

	Coefficient (95% CI) (*p*-value)^a^	
**Contrast**	**Day 21 vs. Baseline** ^b^	**Day 42 vs. Baseline**
*mITT analysis*		
Standard-dose vs. placebo	0.13 (-0.16, 0.42) (*p* = 0.39)	0.35 (0.06, 0.64) (*p* = 0.02)
High-dose vs. placebo	-0.12 (-0.41, 0.16) (*p* = 0.40)	-0.08 (-0.37, 0.20) (*p* = 0.57)
Either dose vs. placebo	-0.001 (-0.26, 0.26) (*p* = 0.99)	0.12 (-0.13, 0.38) (*p* = 0.35)
*Age-adjusted analysis*		
Standard-dose vs. placebo	0.12 (-0.17, 0.41) (*p* = 0.4)	0.34 (0.05, 0.63) (*p* = 0.02)
High-dose vs. placebo	-0.12 (-0.41, 0.16) (*p* = 0.4)	-0.08 (-0.37, 0.20) (*p* = 0.57)
Either dose vs. placebo	-0.003 (-0.26, 0.25) (*p* = 0.98)	0.12 (-0.14, 0.38) (*p* = 0.36)

CI, confidence interval; IgG, immunoglobulin G; mITT, modified intention to treat.

^a^ Between-group differences at follow-up visits estimated using repeated measures linear regression with a random effect for time and represented by marginal means with 95% CIs.

^b^ Primary outcome assessment occurred at Day 21

#### Secondary outcomes

Plasma IgA samples were negative for all subjects at all timepoints. Among nasal washes, only two samples from the same subject in the high-dose group were positive to IgA at D21 and D42.

**Fig. 3:**
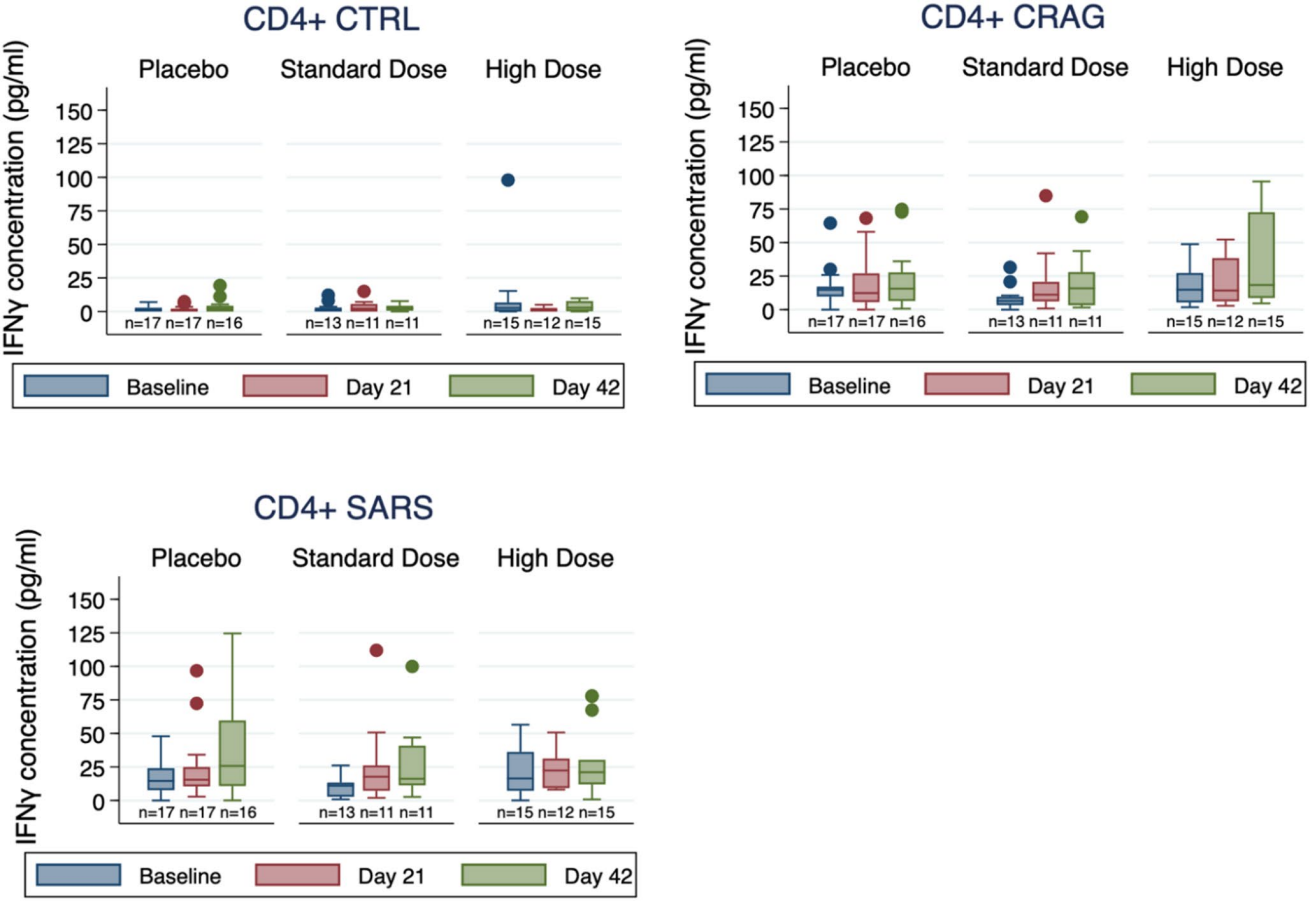
Change in IFNg concentration, CD4+-specific T-cell peptides. CRAG cross-reactive antigen pools; CTRL, control peptides pool; SARS, SARS-CoV-2 peptides pool. Box plots of IFNg concentrations with stimulation by CD4-specific CRAG, CTRL, and SARS peptide pools, stratified by treatment group (Placebo, Standard Dose, and High Dose) and timepoint (baseline, blue; Day 21, red; Day 42, green). Box plots show 25th percentile (lower box line), median (middle box line) and 75th percentile (upper box line). Whiskers show upper and lower adjacent values. Outside values are shown as separate points

**Fig. 4:**
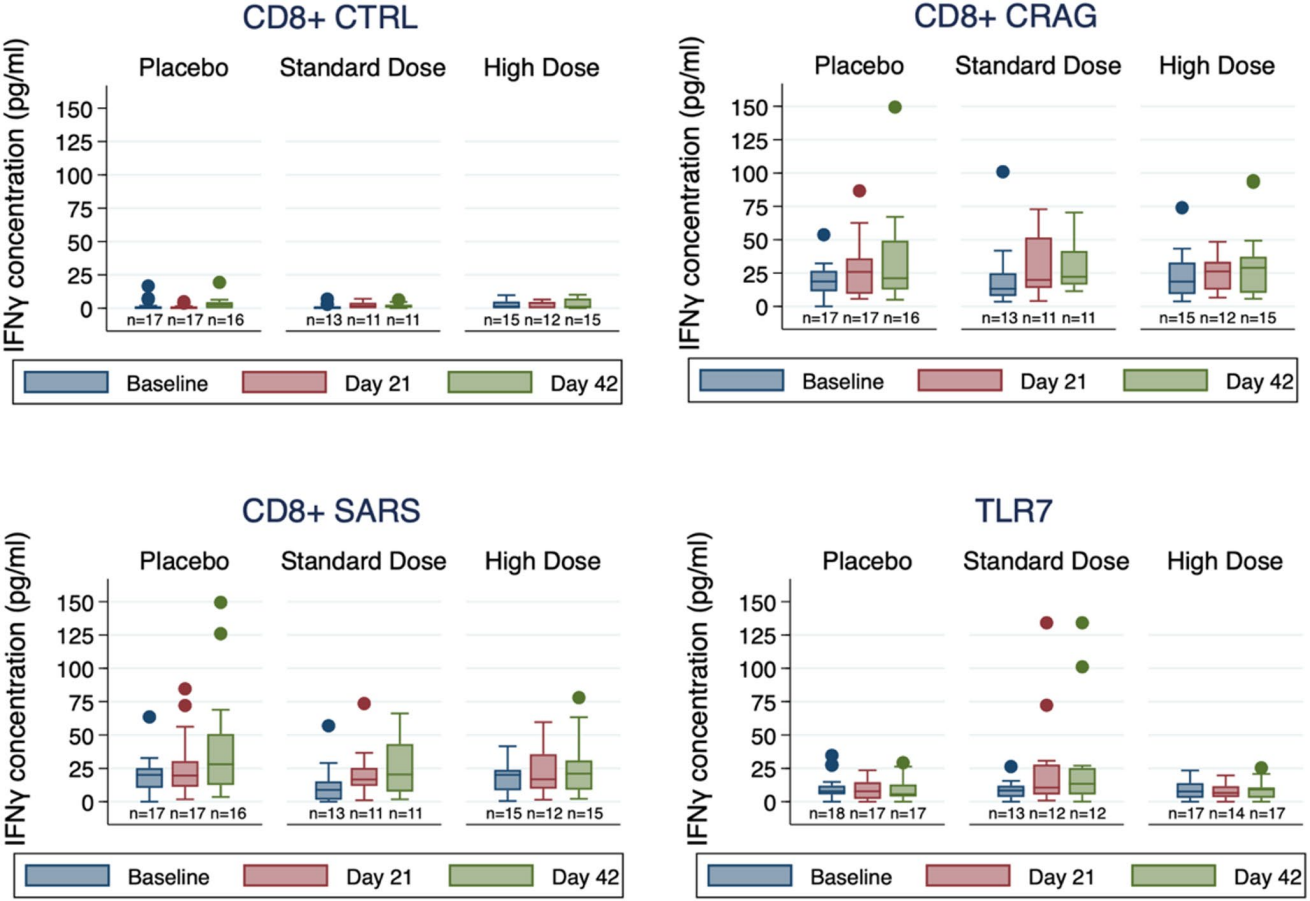
Change in IFNg concentration, CD8+-specific T-cell peptides and TLR7 ligand. CRAG, cross-reactive antigen pools; CTRL, control peptides pool; SARS, SARS-CoV-2 peptides pool. Box plots of IFNg concentrations with stimulation by CD8-specific CRAG, CTRL, and SARS peptide pools or TLR7 ligand (R837), stratified by treatment group (Placebo, Standard Dose, and High Dose) and timepoint (baseline, blue; Day 21, red; Day 42, green). Box plots show 25th percentile (lower box line), median (middle box line) and 75th percentile (upper box line). Whiskers show upper and lower adjacent values. Outside values are shown as separate points

No significant changes in IFNg levels were observed after stimulation of PBMCs with CD4^+^-specific peptides (Fig. 3, Supplementary Table 3). Marginal numeric increases in IFNg levels were seen in the standard-dose group after stimulation with CD4+-specific SARS-CoV-2 peptides (Day 21) and CD4^+^-specific peptides derived from potential CRAGs (Days 21, 42). A similar pattern was observed when examining relative changes in IFNg levels by group (Supplementary Table 4). No substantive increases were observed after stimulation with CD8^+^-specific peptides or R837 (imiquimod), a ligand for TLR7, an innate immune receptor for single-stranded RNA (Fig. 4, Supplementary Table 3), although relative changes in IFNg after TLR7 ligand stimulation were significantly higher in the standard-dose group at Day 21 compared to the other groups (Supplementary Table 4).

Levels of plasma antibodies against probiotic bacterial antigens (e.g., in Bl-04, Bi-26, Lpc-37) were variable across the population, and within-individual levels clustered together across timepoints (Supplementary Fig. 2). While some differences were observed in strain-specific anti-probiotic antibodies in the standard- and high-dose groups relative to placebo, few differences were statistically significant (Supplementary Table 5). Significant decreases in anti-Ls-33 antibodies were seen in the standard-dose group compared to placebo at Day 21 (IgM) and Day 42 (IgA).

While only 7 participants endorsed having PASC, most participants (all but one) reported having at least one symptom of prior SARS-CoV-2 infection at baseline (Table 1). Most participants experienced overall improvement in symptoms during the trial, without major differences observed across groups (Supplementary Table 6). No new (repeat) SARS-CoV-2 infections were detected at any study visit or reported by participants through weekly questionnaires.

### Safety

Most observed AEs were mild in severity, affecting 9 (50%) in the placebo group and 25 (74%) in active treatment groups (*p* = 0.13) (Supplementary Table 7). The most common AEs were gastrointestinal symptoms (40%), including abdominal pain (27%) and diarrhea (21%), and pain (40%), particularly headaches (27%). Overall, gastrointestinal, respiratory, and pain AEs tended to occur more often among participants receiving the active investigational products than among participants receiving placebo, especially headaches (38% vs. 6%, *p* = 0.02). No serious AEs were observed. Vital signs were generally stable during follow-up (Supplementary Table 8).

## Discussion

In this pilot study of giving the live microbial consortium OL-1 to unvaccinated, previously SARS-CoV-2-infected participants, a potential signal for efficacy from OL-1 was observed, with a slight increase in anti-SARS-CoV-2 IgG seen in the standard-dose groups relative to placebo during the study period. This increase was statistically significant at Day 42, 21 days after active treatment was stopped; we did not see any significant differences at the primary endpoint assessment on Day 21. These serologic changes were accompanied by marginal numeric increases in IFNg release after stimulation with CD4^+^-specific CRAG peptides, SARS-CoV-2 peptides, and TLR7 ligands; most of these changes were not statistically significant. While these results were limited by the small number of participants and absence of documented repeat SARS-CoV-2 infections, they suggest that standard-dose OL-1 could possibly enhance clinical protection against recurrent SARS-CoV-2 infection. Confirmation of the effects of OL-1 would require study in a larger, more generalizable population.

Multiple RCTs have investigated the potential for probiotics, prebiotics, or synbiotics to combat COVID-19, including treatment of patients hospitalized with COVID-19, treatment of SARS-CoV-2-infected outpatients, post-exposure prophylaxis for people with household exposures, adjunctive therapy for people receiving COVID-19 vaccination, and treatment for long Covid [32–34]. Among studies examining primary or secondary prevention of COVID-19, several studies have had signals consistent with potential benefit. Two RCTs investigated the effect of distinct probiotics (one yeast-based, the other a *Loigolactobacillus coryniformis* strain) in two different adult populations of COVID-19 vaccine recipients, but neither showed significant differences in serologic or T-cell responses with probiotic exposure [35, 36]. However, in a subgroup of 18 SARS-CoV-2-infected older adults in the larger trial, probiotic-treated participants had higher IgG levels [36]. In other placebo-controlled RCTs that tested the effect of probiotics or synbiotics in high-risk persons (healthcare workers or people with SARS-CoV-2-infected household members), a numerically lower incidence of COVID-19 was seen in two of three trials: a trial of a synbiotic performed with 60 healthcare workers (0% synbiotic vs. 9.7% placebo, *p* = 0.24) [37], and a trial of *Lacticaseibacillus rhamnosus* GG performed with 182 adults with household COVID-19 exposures (8.8% probiotic vs. 15.4% placebo, *p* = 0.17) [16]. In the latter trial, probiotic recipients were significantly less likely to report symptoms 4 weeks after enrollment (26.4% vs. 42.9%, *p* = 0.02) [16]. However, neither these trials nor another showed significantly lower COVID-19 incidence with probiotic or synbiotic treatment [38]. 

The 5 live microbial strains in the OL-1 consortium tested in the Live BASIC Trial were selected based on the presence of CRAGs related to SARS-CoV-2 antigens as well as evidence of antiviral cytokine release following cell stimulation in vitro and potential efficacy in a ferret model of SARS-CoV-2 infection [17]. The findings of the Live BASIC Trial lend some support to the hypothesis that exposure to OL-1 may help stimulate humoral immunity against SARS-CoV-2 in previously infected persons. Other studies have shown that differences in anti-spike IgG level correspond to changes in subsequent infection risk. For instance, in a large prospective cohort of unvaccinated young adults from 2020, those with higher anti-RBD antibody levels had significantly lower rates of subsequent reinfection across several antibody levels (adjusted hazard ratio 0.67, 95% CI 0.47–0.96) [39]. In another prospective cohort of mostly vaccinated individuals (12% with documented SARS-CoV-2 infections in the prior year) followed in 2021–2022, higher anti-spike antibody levels were associated with lower rates of subsequent infection [40]. In another population-based cohort followed in 2020–2021, higher anti-spike antibody levels were also associated with lower hazards of subsequent infection—findings that only applied to those with prior infections, irrespective of vaccination history [41]. Nonetheless, generalizing from these other studies to our own is somewhat complicated by differences in the populations and the methods used for immune assessment. The serologic differences in our study were also relatively small: we observed just a two-fold increase in anti-RBD IgG titer in the standard-dose group relative to the placebo group. With respect to cellular immunity, while we observed several numeric increases in IFNg release from T-cell-specific peptides (SARS-CoV-2-specific and CRAGs) in the standard-dose group relative to placebo, these changes were not fully congruent with the serologic findings and did not reach statistical significance. We also did not find compelling evidence that levels of plasma antibodies against probiotic bacterial antigens corresponded with the observed serologic changes. Collectively, these small and borderline findings do not suggest that OL-1 induces robust immunologic changes but still leave unanswered questions about the clinical significance of its effects that warrant further study.

Another finding that is challenging to interpret is the apparent differential effects of OL-1 between the two active treatment arms. One explanation for this difference is the presence of a non-linear dose-response relationship in serologic response. Of note, not all observed immune effects were distinct between active treatment arms; compared to those in the placebo group, OL-1 treated participants in both arms had relatively higher levels of IFNg production in response to stimulation with CD4+-specific CRAG peptides. However, these findings emerged in the context of multiple secondary analyses and so should be regarded cautiously. Differences between the active treatment arms could also reflect the small sample size of this pilot study, which was not powered to detect small differences; thus, either the increase in the standard-dose group or the lack of change in the high-dose group could theoretically have reflected a chance finding that might differ with a larger sample. By chance, the group randomized to the standard dose was significantly younger than either other group. It is unclear whether other unmeasured differences might have also explained the differences in immune responses observed, although adjustment for age alone changed neither the findings nor conclusions. It is also possible that mild or asymptomatic SARS-CoV-2 infections occurred in some participants in the standard-dose arm, although no infections were reported or detected during the trial. Additionally, we did not observe probiotic-associated increases in anti-probiotic antibodies that would have suggested adaptive immune mechanisms targeting the bacterial antigens. Further investigation is warranted to understand the biological and clinical significance of the findings observed in the Live BASIC Trial.

OL-1 was safe and well tolerated in the study population, with mostly mild AEs, most commonly gastrointestinal AEs. Headaches, gastrointestinal, and respiratory symptoms tended to occur more often in OL-1-treated participants. Headaches have not been a commonly reported AE in prior trials of the strains in OL-1. It is unclear whether the excess of AEs could have resulted not from the treatment but from unexpectedly higher rates of mild infections in the treatment groups.

The Live BASIC Trial had many strengths. Despite targeting a narrowing population of previously (but not recently) infected persons who were not vaccinated, study enrollment was reached on time and exceeded the intended target of evaluable participants, aided by expansion of enrollment to additional geographic areas after initiation. Retention and adherence of participants to study procedures also was excellent, with no loss to follow-up and only one participant excluded at the final study visit because of COVID-19 vaccination. These strengths attest to the potential benefits of fully decentralized trials, including in-person visits conducted directly in participants’ homes. Furthermore, the trial tested several aspects of participants’ immune response, both systemic and mucosal, humoral and cellular, to yield a more complete picture about the potential impact of OL-1 on anti-SARS-CoV-2 immunity.

A major limitation of this pilot study was the small sample size, which limited the ability to detect significant differences between arms and did not permit examination of the clinical significance of immune effects, such as the risk of reinfection, or the impact on symptoms or sequelae of prior SARS-CoV-2 infection. Related to the small sample size, even after randomization, there was chance imbalance among groups in certain characteristics, including age and, to a lesser extent, characteristics of prior COVID-19, which may have impacted the findings in ways that were not wholly apparent or addressable through adjustment alone. The conduct of multiple secondary analyses in evaluation of both efficacy and safety could have led to chance findings. We did not conduct additional subgroup analyses (e.g., by COVID-19 severity or time since infection) because of the small sample size and minor differences observed, to limit the likelihood of chance findings. We also lacked information on variants from prior infections. The study population was not particularly racially or ethnically diverse, a potential benefit of decentralized trials, but one not realized in our trial [42]. Additionally, the inclusion of generally healthy adults 60 and younger may limit our study’s generalizability to other populations, including older adults and people with serious comorbidities, who may be more susceptible to severe COVID-19.

## Conclusion

A 3-week course of treatment with the standard dose of the novel live microbial consortium OL-1 in previously infected, unvaccinated adults was associated with a slight increase in anti-SARS-CoV-2 immunity in participants. OL-1 was generally well tolerated, with participants taking OL-1 experiencing more headaches, gastrointestinal, and respiratory symptoms, which were generally mild in severity. Further study is warranted to examine whether OL-1 enhances anti-SARS-CoV-2 immunity and clinical protection in both unvaccinated and vaccinated persons.

## Supplementary Information

Below is the link to the electronic supplementary material.

Supplementary Material 1

## Data Availability

The data that support the findings of this study are not openly available due to reasons of sensitivity and are available from the corresponding author upon reasonable request. Data are located in controlled access data storage at Rutgers University.
