# Supplementary material for: Live microbials to boost Anti-SARS-CoV-2 immunity clinical trial (Live BASIC trial): a triple-blind randomized controlled trial
Source: Infection. 2025 Nov 26;54(1):473–85. doi: 10.1007/s15010-025-02697-4 (PMC12864199; doi:10.1007/s15010-025-02697-4)
Supplement: Supplementary file 1 — Supplementary Material 1 [file 15010_2025_2697_MOESM1_ESM.docx]

Supplement to Horton DB, *et al*. "Live Microbials to Boost Anti-SARS-CoV-2 Immunity Clinical Trial (Live BASIC Trial): a Triple-blind Randomized Controlled Trial"

Article Name: Live Microbials to Boost Anti-SARS-CoV-2 Immunity Clinical Trial (Live BASIC Trial): a Triple-blind Randomized Controlled Trial

Journal Name: Infection

Author Names: Daniel B. Horton, Rahul Ukey, Abhilasha Madhvi, Tracy Andrews, Veenat Parmar, Nancy Reilly, Sanna M. Mäkelä, Jonathan Peterson, Leah Hustad, Gloriana Wong, Emily S. Barrett, Natalie Bruiners, Jeffrey L. Carson, Kylie Getz, Patricia Greenberg, Alicia Iizuka, Jason Roy, Alexander W. Pastuszak, Markus J. Lehtinen, Martin J. Blaser, Reynold A. Panettieri, Jr., Maria Laura Gennaro

Corresponding Author:

Daniel B. Horton, MD, MSCE

Center for Pharmacoepidemiology and Treatment Science

Institute for Health, Health Care Policy and Aging Research

112 Paterson Street

New Brunswick, NJ 08901

daniel.horton@rutgers.edu

**SUPPLEMENTARY METHODS**

**Study Overview**

The Live Microbials to Boost Anti-SARS-CoV-2 Immunity Clinical Trial (Live BASIC) Trial was designed as a decentralized pilot, parallel-group randomized controlled trial of two doses (standard and high dose) of the live microbial consortium OL-1 versus placebo, taken daily for 21 days. Randomization occurred prior to the baseline visit (**Supplementary Fig. 1**). The Live BASIC Trial was conducted as a triple-blind clinical trial to limit the influence of unconscious or conscious bias on the conduct and interpretation of the trial and its findings. Participants, investigators, and study staff involved in the conduct and evaluation of participants and data were not aware of the product assignment until after the completion of primary analyses and specified unblinding procedures. The full trial protocol is available upon request.

**Study Population**

Inclusion Criteria:

- Healthy adults (aged between 18 to 60) in good general health as judged by the Principal Investigator based on self-reported medical history.
- SARS-CoV-2 infection >4 months prior confirmed by a positive PCR, antigen test, or COVID-19 antibody test. Acceptable documentation of a qualifying COVID-19 antibody test includes positive testing performed >4 months before enrollment or healthcare practitioner-documented illness consistent with COVID-19 >4 months before enrollment plus a positive antibody test any time before enrollment.
- Body mass index (BMI) between 18.5-39.9 kg/m^2^.
- Agreement to comply with the protocol and study restrictions.
- Access to internet in addition to willingness and ability to use internet-based questionnaires.
- Availability for all study visits.

Exclusion Criteria:

- Recent infection with COVID-19 (based on newly positive PCR or antigen test) within the past 4 months.
- Prior receipt of antibody therapies (convalescent or monoclonal) towards COVID-19.
- Prior receipt of any vaccine against COVID-19.
- Planned receipt of a vaccine against COVID-19 in the following month.
- Prior or current participation in a clinical trial of vaccines against COVID-19.
- Regular (e.g., ≥3 days per week) use of any live microbial (probiotic) supplements in the last 3 months.
- Any acute or chronic respiratory tract disease besides mild to moderate asthma.
- Any diagnosed immunodeficiency.
- Current use of immunosuppressive drugs.
- Any diagnosis of chronic gut disease, such as inflammatory bowel disease or irritable bowel syndrome.
- Morbid obesity (BMI ≥ 40).
- Underweight (BMI < 18.5).
- Current pregnancy or breastfeeding.
- Recent use of antibiotic therapy (within last 4 weeks prior to visit 1).
- Current self-reported addiction to alcohol, drugs, or medications.
- Any condition or reason that makes a participant unsuitable for enrollment, in the Principal Investigator’s judgement (e.g., poor adherence to prior study procedures, chronic use of another food supplement or product containing live microbials (probiotics) at more than 10x10^9^ CFU)
- Non-English speaking

**Investigational Product**

Standard dose OL-1 (total at least 43x10^9^ colony forming units (CFU), batch 1103894025), high dose OL-1 (total at least 75x10^9^ CFU, batch 1103868193), and placebo (batch 1103868191) were formulated as capsules containing potato maltodextrin, silicon dioxide, and magnesium stearate. The high dose OL-1 product consisted of equal numbers (approximately 15x10^9^ CFU) of each of the five microbial strains. The standard dose OL-1 product consisted of higher numbers of certain strains (approximately 20x10^9^ CFU of Lpc-37 and Bl-04) and lower numbers of the remaining strains (approximately 1x10^9^ CFU of Bi-26TM, Lr-32®, and Ls-33®).

**Treatment Assignment**

Prior to initiating enrollment, two randomization tables for assignment to one of three study groups were generated by the investigational product manager at IFF Health & Biosciences with nQuery (GraphPad Software DBA Statistical Solutions, MA, USA) and provided via password-protected file to the research pharmacist. Randomization tables were made available to a physician and a back-up pharmacist at Vault Health in case of emergency unblinding, but these individuals did not have any regular contact with participants and never needed to access the tables. Each randomization table consisted of 4-digit randomization codes organized in permuted blocks of six and could accommodate up to 78 participants. One table was intended for selected participants who lived within New Jersey, New York, or Pennsylvania to accommodate direct shipment of blood specimens to the research laboratory of Dr. Maria Laura Gennaro for processing, storage, and preliminary investigation of peripheral blood mononuclear cells (PBMCs) from this trial subset. The other table was intended for use for the remaining participants who lived elsewhere in the US. However, before enrollment of the first participant, the team established that the central repository could process and store PBMCs for all study subjects irrespective of participants' state of residence. Thus, the logistics of the trial were changed such that all samples would be shipped first to the central repository for processing and storage. Because of this, only a single randomization table was used for group assignment for the entire study population without stratification.

The research pharmacist prepared kits containing the 21-day supply of investigational products (live microbials or placebo), which were sequentially numbered in accordance with the randomization tables. The contents of each kit were indistinguishable. Participants were instructed to take the investigational product daily with breakfast along with water (tap, filtered, bottled, or still) or milk. Participants were asked not to take the investigational product with hot drinks, such as coffee or tea, or with alcohol.

Participants had bottles of investigational product labeled with their identification number, and the contents of the investigational product of all three treatment arms without specification of their own assigned treatment. The capsules for each treatment arm were indistinguishable from one another in size, shape, color, taste, and smell. The specific microbial strains of OL-1 were not identified on the label. The research pharmacist securely maintained the master list of treatment assignments without sharing with other team members until after analyses, or in case of emergency unblinding.

Of note, laboratory colleagues in the biorepository and Dr. Gennaro's lab remained blinded to group assignment during the conduct of all assays.

Participants remained blinded to their assigned product until after acceptance of the primary study results for publication.

**Efficacy Outcomes**

Immunologic outcomes:

1. Plasma anti-SARS-CoV-2 IgG titer (change from baseline to Days 21 and 42)

The enzyme-linked immunosorbent assay (ELISA) targeting the Receptor Binding Domain (RBD) of the S1 subunit of the viral spike protein was established and used in the laboratory of Dr. Gennaro, accurately measures immunity after SARS-CoV-2 infection and is comparable to commercially available assays.^1^ End-point titers were established for each sample based on serial plasma dilutions and background-subtracted data.

1. Plasma anti-SARS-CoV-2 IgA antibody (change from baseline to Days 21 and 42)

Serum/plasma anti-SARS-CoV-2 IgA titer represents another important measure of viral immunity that has different kinetics following SARS-CoV-2 infection compared to serum/plasma IgG. Like plasma IgG, anti-SARS-CoV-2 IgA was assessed by ELISA to the RBD portion of the viral spike protein in the laboratory of Dr. Gennaro.^1^

1. Nasal anti-SARS-CoV-2 IgA antibody (change from baseline to Days 21 and 42)

Nasal wash anti-SARS-CoV-2 IgA titer represents an important measure of mucosal immunity to SARS-CoV-2. This antibody was measured within a sample of saline infused and then extracted from the nostril (nasal wash). Like plasma IgG, nasal wash anti-SARS-CoV-2 IgA was assessed by ELISA to the RBD portion of the viral spike protein using an assay adapted for the Live BASIC Trial in the laboratory of Dr. Gennaro.

1. Cytokine release (IFNγ) in response to stimulation of PBMCs with SARS-CoV-2 peptides (change from baseline to Days 21 and 42)

All known T-cell epitopes identified across the SARS-CoV-2 genome curated in the IEDB database with positive T-cell assay data were compared to putative expressed genes contained in probiotic OL1 consortia genomes. All probiotic gene segments sharing greater than 50% homology to SARS-CoV-2 T-cell antigens were analyzed for MHC class I or class II binding probabilities using the IEDB MHCI and MHCII binding prediction tools. OL1 peptides sequences with high SARS-CoV-2 homology and predicted to bind the same HLA alleles in the same binding register as the corresponding SARS-CoV-2 antigen (cross-reactive antigens, CRAGs) were selected for synthesis. Matched control peptides were selected by analyzing the full parent protein sequence containing the putative cross-reactive peptide and selecting a non-homologous sequence predicted to bind with similar affinity and HLA allele profile as the matched cross-reactive peptide.

**CD4 15mer peptides**

| **CD4 SARS-CoV-2 epitope ID#** | **SARS-CoV-2 peptide** | **CRAG peptide** | **CRAG control peptide** |
| --- | --- | --- | --- |
| 1 | IILFLALITLATCEL | IIYFLALITVSTSLL | SFSTAFFTAVSALST |
| 2 | LMIERFVSLAIDAYP | LGIERTVSLARRAWG | KVQLLTLFSTMLGIE |
| 3 | AADLDDFSKQLQQSM | EFGLDDFLAQLQQVR | LQQVRKLGSMKSLLG |
|  |  | AADGWDLDSQLEQAM | YGDRVILDDVTLSFL |
|  |  | SFGLDDFLDQLQQVR | YDTVIIDTAGRLGVD |
|  |  | PADLDDIKTELQQEG | QNRYRTLLPGATYRQ |
| 4 | DAALALLLLDRLNQL | DIELAALELDRLHQA | YDVLQLLKGFGTYIH |
| 5 | KVTFFPDLNGDVVAI | KPSFFPDLLEDVRAQ | YFYDQLAAASSVPLL |
| 6 | TFKCYGVSPTKLNDL | VFKDYQVSHTKMRDL | RHQYVLDFSNPAVVN |
| 7 | MFVFLVLLPLVSS | TFLILVLIPLTSS | GGSRSLLAMGNLLAP |
| 8 | EFYAYLRKHFSMMIL | EFYAYISTGVIMMIL | EVMKPAYILMLGRVV |
| 9 | NKDGIIWVATEGALN | NNSKIIAVATGGSLN | IILGVLLILLLAYLS |
| 10 | NLLLQYGSFCTQLNR | VLVLMIGSFCTILNA | SFIAPNFATLLAGRL |

**CD8 9mer peptides**

| **CD8 SARS-CoV-2 epitope ID#** | **SARS-CoV-2 peptide** | **CRAG peptide** | **CRAG control peptide** |
| --- | --- | --- | --- |
| 1 | DTDFVNEFY | DVDFRNELY | VLDSQRWYY |
|  |  | DTDIVNDIY | ITDQINKVY |
| 2 | LTDEMIAQY | TTNEMIAQY | RADFFSLQL |
| 3 | ALWEIQQV | ALWEIGAVV | FLTGDLGYL |
| 4 | FIAGLIAIV | FISGVIAIV | ILVSFISGV |
| 5 | FLLNKEMYL | FLLNKEIYR | KMMLGILRA |
| 6 | FLNRFTTTL | RLLRFTTTL | QLADALAEI |
| 7 | GLTVLPPLL | GLTILPELL | VVFGGMGSL |
| 8 | KLWAQCVQL | KLGAQWVQL | SLYDQMLDT |
| 9 | LLYDANYFL | LLYLANYNV | ALDSGELDL |
|  |  | LMIDVNYFL | SLLKYSIPL |
|  |  | LLYDANDLA | LLFRINMSV |
|  |  | LLYSATSFL | LMMVASNEV |
| 10 | RLDKVEAEV | RLDKVDALV | LVANGLIAV |
| 11 | SIIAYTMSL | GIDAYTMSL | YLLDVLGEV |
|  |  | TILAYSMSL | FTLAIILPV |
|  |  | TIFAYSMSL | GLTGALFFV |
| 12 | TLGVLVPHV | TLGVLPPTV | VLAEHSAWV |
| 13 | TLMNVLTLV | TLMNVLSGV | ALNDVNIMV |
|  |  | SLNNVLTLV | FMPGVLLAV |
| 14 | VLNDILSRL | SLNDELSRL | TMWIPPDSV |
| 15 | ALSKGVHFV | ALSKGVRIV | QLMGELSDI |
|  |  | WIAGVVAIV | VMFSLTGFV |
| 16 | FIAGLIAIV | FIASVICIV | ALRETVMSA |
| 17 | GMSRIGMEV | GIIRIGAEV | ILAEKPGVV |
|  |  | RLNEESSNL | FVRGYTSTV |
| 18 | RLNEVAKNL | RLMEVTKNA | ILAGMHIGL |
|  |  | RLSEVFNNL | LLSKHVKSV |
| 19 | YLATALLTL | YLATVVLTG | MIAGRSTAM |
| 20 | LALLLLDRL | QALLFLDRL | GVDNRPVAV |
| 21 | LLLDRLNQL | LILDHLHQL | LTIAILTAL |
|  |  | ILLDGLQQL | ALIEEGTPV |
| 22 | LQLGFSTGV | LQLGFSRGL | LQGSWLGPI |
|  |  | LQLGASTGV | FAIAIGLYV |
| 23 | LVLSVNPYV | LVLSVNGYL | YLLGLLPAI |
| 24 | ALRANSAVK | ALQANAAVK | IIGASGSGK |
| 25 | ASAFFGMSR | AVAFFLMSR | RQQAIFFRK |
| 26 | GTITVEELK | GTITVHELR | SAIRIFLTR |
| 27 | KTFPPTEPK | ETFDPTKPK | YTDNVPLLK |
|  |  | KTPAPTTPK | GLANFKFYR |
| 28 | VYIGDPAQL | VYLGDQARL | HYPILRPLI |
| 29 | YYQLYSTQL | YYQLYTGSL | PYTVFVLVF |
|  |  | YYQRHSTRL | HWQASTANF |
| 30 | FPRGQGVPI | KPLGGGVPI | VPMSYEAAL |
|  |  | FPRGRIVEI | APGRETASV |
|  |  | MPQWQGVPI | MPTYPAATM |
|  |  | FVRGQGVTV | IPGKPVSEV |
| 31 | IPRRNVATL | IPRQNVVTV | RIRGVSKSF |
| 32 | SPRRARSVA | SVRRARAVA | LPAHGESVL |
| 33 | GEAANFCAL | VEAANDCAL | SENRGLITV |
|  |  | GQAANFFAL | GESISNPAL |
| 34 | GETLPTEVL | GETLPIEHL | RETETGLIL |
|  |  | GEALPTLVL | WEDMAKSGI |
| 35 | LEPLVDLPI | LERLADLPI | VEDQDHPSV |
| 36 | MEVTPSGTW | MEQTKDGTW | KEALDAIHF |
| 37 | CTDDNALAY | VLDDNHLAY | ISEIEDLSA |
|  |  | ITDENAPAY | RTDNWFLLA |
|  |  | STDDRAAAY | CTGHGNGLY |

PBMCs were maintained in pre-warmed complete RPMI (RPMI 1640 supplemented with 2 mM l-glutamine, 10% fetal bovine serum, 100 U/ml penicillin, and 100 μg/ml streptomycin (Corning cellgro, Manassas, VA, USA), stimulated with a SARS-CoV-2 peptide pool, CRAG pool, and control peptide pool (CD4+ and CD8+) at 4 µM final concentration or 0.1% DMSO control, utilizing standard procedures.^2^ PBMCs were also stimulated with R837 (TLR7 ligand) (InvivoGen, San Diego, CA, USA) at 5µg/ml final concentration. Supernatants were collected after 24 hours and tested for IFNγ using an ELISA kit (BD Biosciences, San Jose, CA, USA), and measured in pg/ml.^1^

1. Plasma antibodies binding probiotic antigens (change from baseline to Days 21 and 42)

Ingested probiotic bacteria may interact with gut mucosal tissues and induce adaptive T-cell and B-cell responses. Treatment-related changes in circulating antibodies that bind probiotic surface antigens could suggest that probiotic antigen sensing in the gut mucosa can influence adaptive immune effects systemically. Freeze dried concentrates of probiotic bacterial cells were resuspended at 25mg/ml tris-buffered saline (TBS) for 20 minutes on a rotational mixer. Cells were diluted 1:100, and 10µl was added to V-bottom microtiter plates. Plasma was diluted 1:1 with TBS, and 10µl was combined with target cells and incubated for 30 minutes at 30°C. Cells were washed three times in 250µl TBS containing 0.2% bovine serum albumin and 2mM EDTA; plates were centrifuged at 3000 x g for 5 minutes to pellet bacteria between washes. Detection antibodies were diluted 1:200 in wash buffer, and 20µl was applied to washed target cell pellets for 30 minutes at 30°C. 100 µl of wash buffer containing 1:10,000 dilution of syto24 dye was added to each well and allowed to incubate for 10 minutes. Fluorescent signals associated with IgG, IgA, and IgM were measured for each sample by flow cytometry.

Clinical outcomes:

1. Change in any baseline long-term symptoms or sequelae of prior SARS-CoV-2 infection

Pre-existing symptoms or sequelae of prior SARS-CoV-2 infection were recorded in baseline questionnaires and tracked through weekly symptoms questionnaires. These symptoms may have included fatigue, altered taste or smell, shortness of breath, or joint pain.

1. Incidence of new (repeat) SARS-CoV-2 infections

SARS-CoV-2 assays were performed under FDA-approved EUA#200090 at Infinity Biologix® (Piscataway NJ), as described.^3,4^ In brief, total RNA was extracted from saliva using nucleic acid-binding paramagnetic beads (Chemagic Viral DNA/RNA 300 Kit H96). Reverse transcriptase-polymerase chain reaction (RT-PCR) was performed in triplicate for three SARS-CoV-2 genomic regions: nucleocapsid (N), spike protein (S), and ORF1ab. Positive and negative assay controls were used.

1. Adverse events and serious adverse events (SAEs)

Adverse events and SAEs were determined from the time the investigational product is given, throughout the period of investigational product use and up to and including the 3-week follow-up period. All AEs were assigned grade 1-3 based on self-reported severity assessment (mild, moderate, or severe). After withdrawal from the investigational product, participants were followed-up for all existing and new AEs for up to 30 days after study completion or until event resolution, whichever was sooner. All new AEs occurring during that period were recorded. SAEs were reported to the IRB as per SOPs. All study-related toxicities/SAEs were followed until resolution unless the condition was judged to be unlikely to resolve due to the patient’s underlying disease.

AEs were assessed individually (e.g., bloating, abdominal pain) and collectively (any AE) by severity (mild, moderate, or severe) and coded by MedDRA using the Common Terminology Criteria for Adverse Events (CTCAE). SAEs were assessed individually (e.g., hospitalization for arrhythmia) and collectively (any SAE). AEs were reported by participants via weekly questionnaires or virtual study visits with the lead coordinator at Vault Health. Causality was assessed by Dr. Pastuszak at Vault Health.

**Unblinding**

After completion of plasma IgG and IgA assays from the final (Day 42) samples for the final randomized participant on study, a database lock was performed, and treatment groups were unblinded as groups A, B, and C, without revealing group assigned to the investigational product. Statistical analyses were performed comparing groups pairwise: A vs. B, A vs. C, and B vs. C as intention-to-treat (primary) and per-protocol (secondary), without knowledge of which of the two comparisons included the placebo group. Following these analyses, assignments to each dose of investigational product vs. placebo were fully unblinded, allowing the team to focus on the prespecified comparisons of active product vs. placebo groups.

**Statistical Analysis**

In linear regression models, biospecimen collection visit number was included as a random intercept, and adjustments were made for autocorrelation within subjects. Marginal means were estimated to contrast values for each study arm across visits.

The per-protocol population was determined separately at each timepoint (e.g., Day 21, Day 42). That is, a subject who was vaccinated at Day 30, without other prior deviations, was analyzed in the per-protocol population for outcomes at Day 21 (including the primary outcome) but not at Day 42.

Two enrolled and randomized participants lacked any available follow-up data and did not initiate the investigational product: one participant (allocated to the standard-dose arm) was withdrawn due to vaccination before the baseline study visit and product initiation; the other participant (allocated to the high-dose arm) was unable to schedule any study visits and, thus, did not provide any biospecimens or start the investigational product. Thus, the final modified intention to treat (mITT) study population consisted of 52 randomized subjects who began taking the investigational product. Of these, a baseline plasma sample was missing for one subject. The efficacy subset was limited to all participants who were randomized and received investigational product minus the participant missing a baseline plasma sample.

There were no adjustments for multiple comparisons in analyses of secondary outcomes. The main source of missing data was missing outcome data from missed or inadequate samples. Available outcome data were incorporated into statistical analyses apart from immunologic data from the participant missing a baseline sample. Otherwise, no additional approaches were used for missing data.

**SUPPLEMENTARY REFERENCES**

1. Datta P, Ukey R, Bruiners N, *et al.* Highly versatile antibody binding assay for the detection of SARS-CoV-2 infection and vaccination. *J Immunol Methods.* 2021:113165.

2. Ukey R, Bruiners N, Mishra H, *et al.* Dichotomy between the humoral and cellular responses elicited by mRNA and adenoviral vector vaccines against SARS-CoV-2. *BMC Med.* 2022;20(1):32.

3. Barrett ES, Horton DB, Roy J, *et al.* Prevalence of SARS-CoV-2 infection in previously undiagnosed health care workers in New Jersey, at the onset of the U.S. COVID-19 pandemic. *BMC Infect Dis.* 2020;20(1):853.

4. Radbel J, Jagpal S, Roy J, *et al.* Detection of Severe Acute Respiratory Syndrome Coronavirus 2 (SARS-CoV-2) Is Comparable in Clinical Samples Preserved in Saline or Viral Transport Medium. *J Mol Diagn.* 2020;22(7):871-875.

**Supplementary Fig. 1 Schematic of the Live BASIC Trial**

**
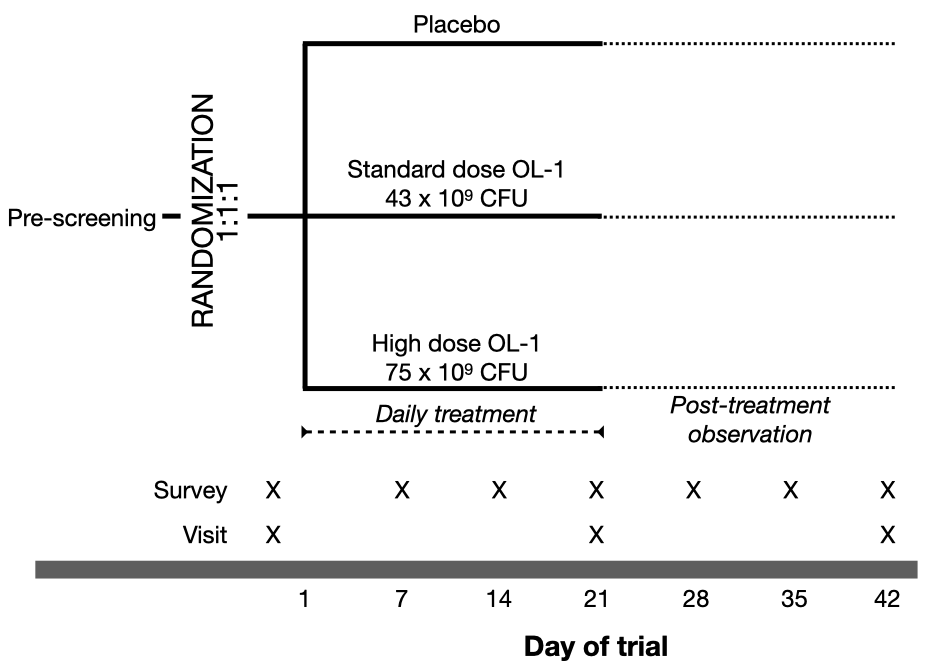
**

CFU, colony forming units. Day 1 was the first day of investigational product. The baseline visit and questionnaire took place before or on Day 1 (prior to receiving investigational product).

**Supplementary Fig. 2 Anti-probiotic antibody levels**

**
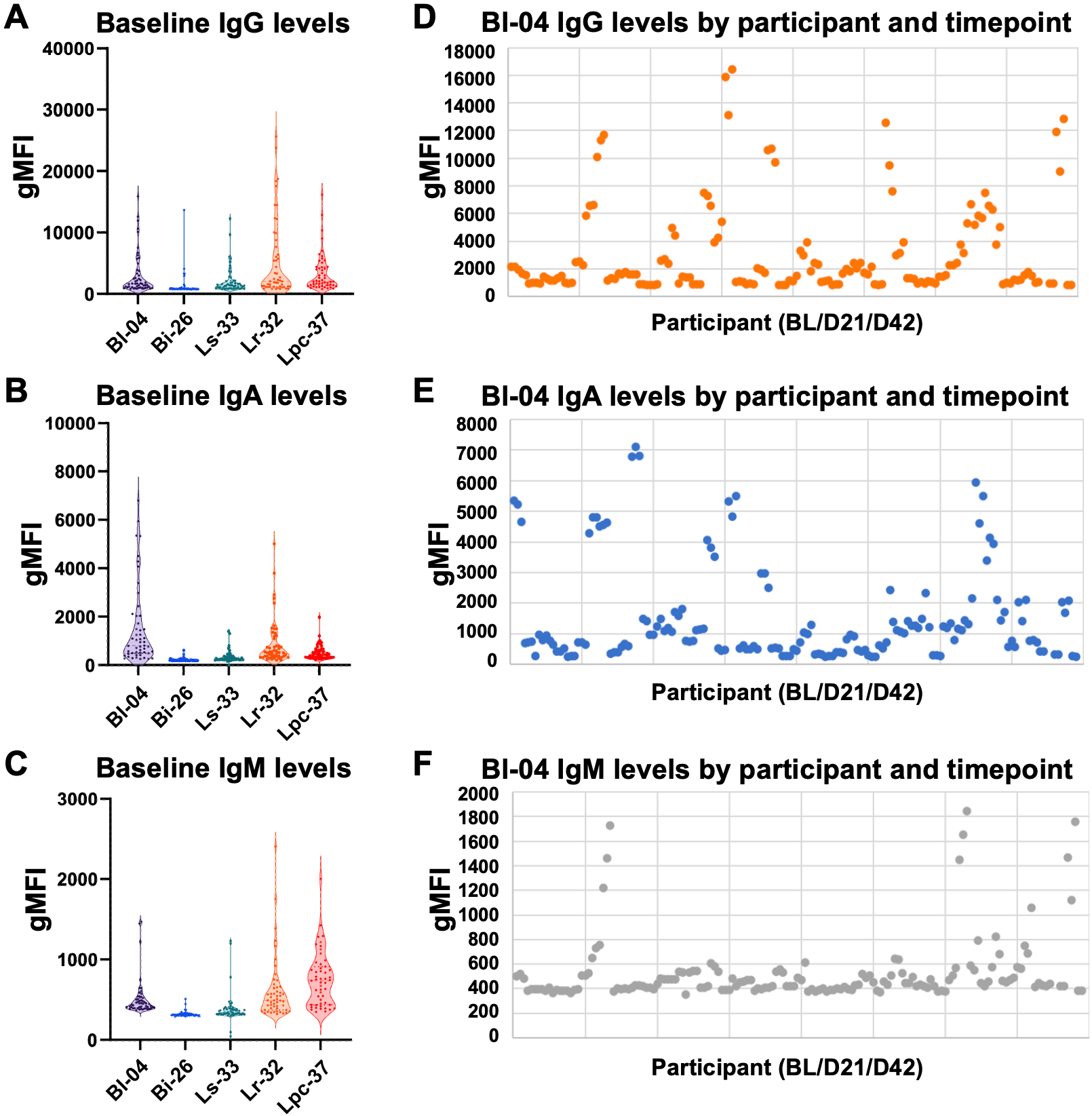
**

Bi-26, *Bifidobacterium longum* subsp*. infantis*; Bl-04, *Bifidobacterium animalis* subsp. *lactis*; gMFI, geometric mean fluorescent intensity; IgA, immunoglobulin A; IgG, immunoglobulin G; IgM, immunoglobulin M; Lpc-37, *Lacticaseibacillus paracasei*; Lr-32, *Lacticaseibacillus rhamnosus*; Ls-33, *Ligilactobacillus salivarius*. Violin plots show baseline levels of plasma IgG (A), IgA (B), and IgM (C) binding antigens from the 5 live microbial strains in OL-1 (note the different y-axes for each plot). Antibody levels against one OL-1 strain, Bl-04, are shown for all participants in arbitrary sequence with 3 adjacent measures per participant representing each consecutive timepoint (baseline, Day 21, and Day 42).

**Supplementary Table 1. Schedule of assessments in the Live BASIC Trial**

| Assessment | Screening | | Baseline | Treatment | | | Follow-up | | |
| --- | --- | --- | --- | --- | --- | --- | --- | --- | --- |
| Study week | -1/-2 | | 0 | 1 | 2 | 3 | 4 | 5 | 6 |
| Informed consent | X | |  |  |  |  |  |  |  |
| History | X | | X |  |  | X |  |  | X |
| Vital signs |  | | X |  |  | X |  |  | X |
| Effectiveness |  | |  |  |  |  |  |  |  |
| Plasma IgG (1°) |  | | X |  |  | X |  |  | X |
| Plasma IgA |  | | X |  |  | X |  |  | X |
| Nasal IgA |  | | X |  |  | X |  |  | X |
| IFNγ release |  | | X |  |  | X |  |  | X |
| Symptoms |  | | X | X | X | X | X | X | X |
| SARS-CoV-2 testing | |  | X |  |  | X |  |  | X |
| Anti-probiotic IgG/A/M | |  | X |  |  | X |  |  | X |
| Safety |  | |  |  |  |  |  |  |  |
| Adverse events |  | | X | X | X | X | X | X | X |
| Vital signs |  | | X |  |  | X |  |  | X |

1° = primary outcome

IFNγ, interferon gamma; IgA, immunoglobulin A; IgG, immunoglobulin G; IgM, immunoglobulin M

**Supplementary Table 2. Number of capsules consumed during the trial**

| **N capsules consumed** | **Total**  **(n=52)**^a^ | **Placebo**  **(n=18)** | **Standard**  **(n=17)** | **High**  **(n=17)** |
| --- | --- | --- | --- | --- |
| 14 | 1 (2%) | 1 (6%) | 0 | 0 |
| 19 | 5 (10%) | 1 (6%) | 3 (18%) | 1 (6%) |
| 20 | 13 (25%) | 4 (22%) | 5 (29%) | 4 (24%) |
| 21 | 24 (46%) | 9 (50%) | 8 (47%) | 7 (41%) |
| 22 | 5 (10%) | 2 (11%) | 1 (6%) | 2 (12%) |
| 23 | 4 (8%) | 1 (6%) | 0 | 3 (18%) |

^a^ Adherence did not significantly differ across groups (Fisher exact p = 0.79)

**Supplementary Table 3. Change in IFNγ concentration by treatment group and stimulation condition**

|  | **Coefficient (95% CI) (p-value)**^a^ | |
| --- | --- | --- |
| **Contrast (mITT analysis)** | **Day 21 vs. Baseline** | **Day 42 vs. Baseline** |
| CD4+ CTRL |  |  |
| Standard-dose vs. placebo | 1.14 (-7.74, 10.02) (p=0.80) | -1.07 (-10.01, 7.86) (p=0.81) |
| High-dose vs. placebo | -7.93 (-16.74, 0.88) (p=0.08) | -7.97 (-16.55, 0.61) (p=0.07) |
| CD4+ CRAG |  |  |
| Standard-dose vs. placebo | 6.63 (-10.35, 23.60) (p=0.44) | 5.40 (-11.65, 22.45) (p=0.54) |
| High-dose vs. placebo | 2.10 (-14.79, 18.98) (p=0.81) | 10.84 (-5.55, 27.23) (p=0.20) |
| CD4+ SARS-CoV-2 |  |  |
| Standard-dose vs. placebo | 10.25 (-8.55, 29.04) (p=0.29) | 0.55 (-18.32, 19.42) (p=0.96) |
| High-dose vs. placebo | 1.03 (-17.67, 19.72) (p=0.91) | -9.07 (-27.22, 9.07) (p=0.33) |
| CD8+ Control |  |  |
| Standard-dose vs. placebo | 2.56 (-0.74, 5.86) (p=0.13) | -0.41 (-3.73, 2.91) (p=0.81) |
| High-dose vs. placebo | 1.82 (-1.45, 5.09) (p=0.28) | -0.19 (-3.37, 3.00) (p=0.91) |
| CD8+ CRAG |  |  |
| Standard-dose vs. placebo | -0.02 (-21.39, 21.34) (p=0.99) | -6.87 (-28.36, 14.61) (p=0.53) |
| High-dose vs. placebo | -5.20 (-26.42, 16.02) (p=0.63) | -4.83 (-25.46, 15.81) (p=0.65) |
| CD8+ SARS-CoV-2 |  |  |
| Standard-dose vs. placebo | 4.48 (-15.68, 24.63) (p=0.66) | -5.81 (-26.06, 14.44) (p=0.57) |
| High-dose vs. placebo | 2.35 (-17.68, 22.38) (p=0.82) | -12.07 (-31.53, 7.38) (p=0.22) |
| TLR7 ligand |  |  |
| Standard-dose vs. placebo | 4.44 (-21.81, 30.68) (p=0.74) | 1.22 (-25.03, 27.46) (p=0.93) |
| High-dose vs. placebo | 1.45 (-23.59, 26.48) (p=0.91) | 0.57 (-23.71, 24.84) (p=0.96) |

CI, confidence interval; CRAG, cross-reactive antigen pools; CTRL, control peptides pool; IFNγ, interferon gamma; mITT, modified intention to treat; SARS, SARS-CoV-2 peptides pool; TLR7, toll-like receptor 7

^a^ Between-group differences at follow-up visits estimated using repeated measures linear regression with a random effect for time and represented by marginal means with 95% CIs

**Supplementary Table 4. Relative change in IFNγ concentration by treatment group and stimulation condition**

| **Stimulation** | **Treatment group** | **Median (IQR) % change, Baseline to Day 21** | **p-value**^a^ | **Median (IQR) % change, Baseline to Day 42** | **p-value**^a^ |
| --- | --- | --- | --- | --- | --- |
| CTRL, CD4+ | Placebo | -31 (-66, 44) | 0.56 | 0 (-65, 57) | 0.17 |
|  | Standard dose | 5 (-22, 64) |  | -21 (-60, 0) |  |
|  | High dose | -63 (-96, -7) |  | 57 (1, 160) |  |
| CRAG, CD4+ | Placebo | -10 (-82, 82) | 0.13 | 0 (-52, 90) | 0.42 |
|  | Standard dose | 87 (21, 170) |  | 120 (-46, 352) |  |
|  | High dose | 53 (-13, 120) |  | 95 (-19, 308) |  |
| SARS, CD4+ | Placebo | -33 (-48, 92) | 0.12 | 2 (-18, 83) | 0.56 |
|  | Standard dose | 120 (-28, 495) |  | 69 (-18, 151) |  |
|  | High dose | 57 (6, 118) |  | 28 (-35, 79) |  |
| CTRL, CD8+ | Placebo | -87 (-100, 7) | 0.18 | 85 (0, 292) | 0.04 |
|  | Standard dose | 53 (-2, 378) |  | -77 (-789, 33) |  |
|  | High dose | 162 (-11, 3350) |  | -62 (-1190, -9) |  |
| CRAG, CD8+ | Placebo | 7 (-43, 94) | 0.73 | -9 (-68, 107) | 0.86 |
|  | Standard dose | 56 (-32, 203) |  | 18 (-75, 169) |  |
|  | High dose | 35 (-33, 150) |  | -16 (-100, 35) |  |
| SARS, CD8+ | Placebo | -22 (-41, 50) | 0.11 | 45 (-0, 105) | 0.24 |
|  | Standard dose | 53 (-6, 154) |  | 44 (-20, 379) |  |
|  | High dose | 30 (-13, 154) |  | 1 (-42, 43) |  |
| TLR7 | Placebo | -40 (-66, 23) | 0.05 | 9 (-38, 53) | 0.89 |
|  | Standard dose | 46 (-10, 363) |  | 15 (-0, 109) |  |
|  | High dose | -11 (-59, 27) |  | 4 (-75, 53) |  |

CRAG, cross-reactive antigen pools; CTRL, control peptides pool; IQR, interquartile range; SARS, SARS-CoV-2 peptides pool; TLR7, toll-like receptor 7

^a^ p-values produced by Kruskal-Wallis testing

**Supplementary Table 5. Comparison of strain-specific anti-probiotic antibodies in the active treatment groups relative to placebo**^a^

| Strain | Day (ref= Day 0) | Dose (ref= Placebo) | IgG Geometric Mean | | IgA Geometric Mean | | IgM Geometric Mean | |
| --- | --- | --- | --- | --- | --- | --- | --- | --- |
|  |  |  | Contrast | P-value | Contrast | P-value | Contrast | P-value |
| Bi-26 | Day 21 | Standard Dose | 148.50 | 0.127 | 1.03 | 0.977 | 0.15 | 0.980 |
|  | Day 42 | Standard Dose | 107.32 | 0.277 | 43.92 | 0.219 | -1.54 | 0.796 |
|  | Day 21 | High Dose | -28.68 | 0.766 | -8.64 | 0.805 | 3.79 | 0.516 |
|  | Day 42 | High Dose | 37.59 | 0.696 | 28.47 | 0.416 | 1.86 | 0.750 |
| Bl-04 | Day 21 | Standard Dose | -97.57 | 0.777 | 55.04 | 0.640 | -7.20 | 0.849 |
|  | Day 42 | Standard Dose | 348.63 | 0.317 | 14.01 | 0.906 | 20.51 | 0.592 |
|  | Day 21 | High Dose | -69.78 | 0.838 | -111.35 | 0.338 | 13.97 | 0.708 |
|  | Day 42 | High Dose | -59.24 | 0.862 | -194.37 | 0.095 | 8.65 | 0.817 |
| Lpc-37 | Day 21 | Standard Dose | 334.05 | 0.152 | 8.09 | 0.809 | 17.09 | 0.711 |
|  | Day 42 | Standard Dose | 59.67 | 0.801 | -33.50 | 0.324 | 9.26 | 0.843 |
|  | Day 21 | High Dose | 108.71 | 0.637 | 34.60 | 0.297 | -8.77 | 0.848 |
|  | Day 42 | High Dose | 99.89 | 0.665 | 20.85 | 0.530 | -42.99 | 0.346 |
| Lr-32 | Day 21 | Standard Dose | -263.19 | 0.727 | -11.03 | 0.919 | 16.34 | 0.702 |
|  | Day 42 | Standard Dose | 489.31 | 0.521 | -157.52 | 0.149 | 1.56 | 0.971 |
|  | Day 21 | High Dose | -919.37 | 0.217 | 70.40 | 0.509 | 14.59 | 0.730 |
|  | Day 42 | High Dose | 185.12 | 0.803 | 45.99 | 0.666 | -17.56 | 0.678 |
| Ls-33 | Day 21 | Standard Dose | 559.94 | 0.308 | -32.84 | 0.389 | -64.59 | 0.023 |
|  | Day 42 | Standard Dose | 109.14 | 0.844 | -120.14 | 0.002 | -28.10 | 0.328 |
|  | Day 21 | High Dose | 159.45 | 0.769 | -11.39 | 0.763 | -16.83 | 0.549 |
|  | Day 42 | High Dose | 132.33 | 0.808 | -13.13 | 0.728 | -8.98 | 0.749 |

Bi-26, *Bifidobacterium longum* subsp*. infantis*; Bl-04, *Bifidobacterium animalis* subsp. l*actis*; IgA, immunoglobulin A; IgG, immunoglobulin G; IgM, immunoglobulin M; Lpc-37, *Lacticaseibacillus paracasei*; Lr-32, *Lacticaseibacillus rhamnosus*; Ls-33, *Ligilactobacillus salivarius*.

^a^ Results were generated from mixed linear regression models evaluating the change between baseline (reference) and Days 21 and 42 in strain-specific antibody level in the standard-dose or high-dose groups compared with the placebo group. Separate models were produced for each live microbial strain and antibody isotype, with group and study day serving as independent variables.

### **Supplementary Table 6. Improvement in symptoms from baseline to Days 1-21 and Days 22-42, treatment groups versus placebo**

|  |  | **Days 1-21 vs. Baseline** | | | | **Days 22-42 vs. Baseline** | | | |
| --- | --- | --- | --- | --- | --- | --- | --- | --- | --- |
| **System** | **Symptom, N (%)** | **Placebo**  **(n=18)** | **Standard Dose**  **(n=17)** | **High Dose**  **(n=17)** | **p-value**^a^ | **Placebo**  **(n=18)** | **Standard Dose**  **(n=17)** | **High Dose**  **(n=17)** | **p-value**^a^ |
| **Gastrointestinal** | Abdominal pain | 5 (36%) | 4 (31%) | 3 (21%) | 0.72 | 5 (28%) | 4 (29%) | 4 (25%) | 0.99 |
|  | Anorexia/weight loss | 13 (72%) | 9 (53%) | 7 (41%) | 0.14 | 12 (75%) | 9 (56%) | 8 (47%) | 0.14 |
|  | Diarrhea | 8 (50%) | 7 (41%) | 3 (21%) | 0.34 | 8 (44%) | 7 (50%) | 3 (18%) | 0.54 |
|  | Nausea/vomiting | 4 (25%) | 3 (19%) | 3 (18%) | 0.71 | 4 (22%) | 3 (20%) | 3 (18%) | 0.99 |
| **Neurologic** | Confusion | 8 (47%) | 8 (50%) | 5 (29%) | 0.76 | 9 (50%) | 8 (50%) | 5 (29%) | 0.56 |
|  | Dizziness | 9 (53%) | 8 (47%) | 5 (29%) | 0.38 | 9 (53%) | 8 (50%) | 5 (29%) | 0.39 |
|  | Loss of smell | 16 (89%) | 12 (71%) | 9 (53%) | 0.06 | 16 (89%) | 12 (71%) | 9 (53%) | 0.06 |
|  | Loss of taste | 14 (78%) | 10 (59%) | 8 (47%) | 0.13 | 14 (78%) | 10 (63%) | 8 (47%) | 0.14 |
|  | Muscle weakness | 10 (59%) | 6 (35%) | 9 (53%) | 0.38 | 9 (50%) | 6 (40%) | 9 (53%) | 0.99 |
|  | Vision/eye issues | 4 (25%) | 2 (12%) | 2 (12%) | 0.25 | 4 (22%) | 2 (13%) | 2 (12%) | 0.44 |
| **Pain** | Headache | 14 (78%) | 14 (88%) | 13 (87%) | 0.44 | 13 (76%) | 14 (82%) | 12 (75%) | 0.99 |
|  | Joint pain | 11 (65%) | 8 (47%) | 9 (56%) | 0.55 | 10 (56%) | 8 (50%) | 9 (60%) | 0.99 |
|  | Muscle pain | 13 (76%) | 10 (63%) | 11 (69%) | 0.53 | 12 (67%) | 10 (63%) | 11 (69%) | 0.99 |
|  | Overall pain | 3 (19%) | 3 (23%) | 3 (25%) | 0.99 | 2 (13%) | 3 (20%) | 3 (23%) | 0.69 |
| **Respiratory** | Chest tightness | 7 (41%) | 5 (29%) | 7 (44%) | 0.77 | 6 (35%) | 5 (33%) | 7 (41%) | 0.99 |
|  | Congestion | 12 (71%) | 14 (88%) | 13 (76%) | 0.48 | 12 (67%) | 13 (76%) | 13 (76%) | 0.52 |
|  | Cough | 12 (67%) | 11 (65%) | 9 (56%) | 0.77 | 12 (67%) | 11 (69%) | 9 (53%) | 0.77 |
|  | Shortness of breath | 8 (47%) | 6 (35%) | 5 (29%) | 0.71 | 7 (41%) | 6 (40%) | 5 (29%) | 0.72 |
|  | Skin tingling/numbness | 4 (24%) | 3 (18%) | 3 (18%) | 0.37 | 4 (24%) | 3 (20%) | 3 (18%) | 0.76 |
|  | Sore throat | 8 (50%) | 6 (38%) | 5 (33%) | 0.37 | 8 (47%) | 7 (47%) | 5 (31%) | 0.76 |
| **Systemic** | Chills | 12 (67%) | 9 (53%) | 9 (53%) | 0.39 | 12 (67%) | 9 (56%) | 9 (53%) | 0.55 |
|  | Fatigue | 17 (94%) | 14 (88%) | 15 (88%) | 0.64 | 16 (94%) | 14 (88%) | 15 (88%) | 0.65 |
|  | Fever | 16 (89%) | 12 (71%) | 11 (65%) | 0.18 | 16 (89%) | 12 (71%) | 11 (65%) | 0.18 |
| **Other** | Anxiety | 2 (13%) | 4 (27%) | 1 (6%) | 0.99 | 2 (12%) | 4 (29%) | 1 (6%) | 0.99 |
|  | Rash | 1 (6%) | 3 (18%) | 2 (12%) | 0.65 | 1 (6%) | 3 (20%) | 2 (12%) | 0.40 |
|  | Problems with usual activities | 3 (19%) | 2 (15%) | 2 (13%) | 0.69 | 2 (12%) | 3 (19%) | 2 (13%) | 0.99 |
|  | Overall symptom severity | 18 (100%) | 15 (88%) | 13 (81%) | 0.15 | 17 (100%) | 16 (94%) | 14 (93%) | 0.54 |

^a^ p-values produced by Fisher exact tests

### **Supplementary Table 7. Summary of adverse events by treatment group**

| **CTCAE** | **Total**  **(n=52)** | **Placebo (n=18)** | **Any IP (n=34)** | **P-value**^a^ |  |
| --- | --- | --- | --- | --- | --- |
|  |  |  |  |  |  |
| **Gastrointestinal** | **21 (40%)** | **5 (28%)** | **16 (47%)** | **0.24** |  |
| Abdominal Pain | 14 (27%) | 4 (22%) | 10 (29%) | 0.75 |  |
| Anorexia | 3 (6%) | 0 (0%) | 3 (9%) | 0.54 |  |
| Bloating | 3 (6%) | 0 (0%) | 3 (9%) | 0.54 |  |
| Diarrhea | 11 (21%) | 3 (17%) | 8 (24%) | 0.73 |  |
| Dyspepsia | 1 (2%) | 0 (0%) | 1 (3%) | 0.99 |  |
| Nausea | 3 (6%) | 1 (6%) | 2 (6%) | 0.99 |  |
| Vomiting | 1 (2%) | 0 (0%) | 1 (3%) | 0.99 |  |
| **Respiratory** | **14 (27%)** | **3 (17%)** | **11 (32%)** | **0.33** |  |
| Allergic Rhinitis | 1 (2%) | 0 (0%) | 1 (3%) | 0.99 |  |
| Cough | 3 (6%) | 0 (0%) | 3 (9%) | 0.54 |  |
| Dyspnea | 1 (2%) | 0 (0%) | 1 (3%) | 0.99 |  |
| Nasal Congestion | 6 (12%) | 1 (6%) | 5 (15%) | 0.65 |  |
| Pharyngitis | 2 (4%) | 0 (0%) | 2 (6%) | 0.54 |  |
| Sore Throat | 4 (8%) | 1 (6%) | 3 (9%) | 0.99 |  |
| **Neurologic** | **7 (13%)** | **3 (17%)** | **4 (12%)** | **0.68** |  |
| Blurred Vision | 1 (2%) | 1 (6%) | 0 (0%) | 0.35 |  |
| Cognitive Disturbance | 1 (2%) | 0 (0%) | 1 (3%) | 0.99 |  |
| Confusion | 2 (4%) | 0 (0%) | 2 (6%) | 0.54 |  |
| Dizziness | 1 (2%) | 0 (0%) | 1 (3%) | 0.99 |  |
| Impaired Concentration | 1 (2%) | 0 (0%) | 1 (3%) | 0.99 |  |
| Olfactory Nerve Disorder | 1 (2%) | 1 (6%) | 0 (0%) | 0.35 |  |
| Paresthesia | 1 (2%) | 0 (0%) | 1 (3%) | 0.99 |  |
| Peripheral Sensory Neuropathy | 0 (0%) | 0 (0%) | 0 (0%) | -- |  |
| **Pain** | **21 (40%)** | **4 (22%)** | **17 (50%)** | **0.08** |  |
| Pain | 2 (4%) | 0 (0%) | 2 (6%) | 0.54 |  |
| Arthralgia | 5 (10%) | 2 (11%) | 3 (9%) | 0.99 |  |
| Back Pain | 1 (2%) | 0 (0%) | 1 (3%) | 0.99 |  |
| Chest Pain | 1 (2%) | 0 (0%) | 1 (3%) | 0.99 |  |
| Ear Pain | 1 (2%) | 0 (0%) | 1 (3%) | 0.99 |  |
| Headache | 14 (27%) | 1 (6%) | 13 (38%) | 0.02 |  |
| Myalgia | 6 (12%) | 2 (11%) | 4 (12%) | 0.99 |  |
| **Systemic** | **12 (23%)** | **3 (17%)** | **9 (26%)** | **0.51** |  |
| Chills | 1 (2%) | 1 (6%) | 0 (0%) | 0.35 |  |
| Fatigue | 9 (17%) | 2 (11%) | 7 (21%) | 0.47 |  |
| Fever | 2 (4%) | 0 (0%) | 2 (6%) | 0.54 |  |
| Malaise | 1 (2%) | 0 (0%) | 1 (3%) | 0.99 |  |
| **Mental Health** | **3 (6%)** | **1 (6%)** | **2 (6%)** | **0.99** |  |
| Anxiety | 3 (6%) | 1 (6%) | 2 (6%) | 0.99 |  |
| Depression | 2 (4%) | 1 (6%) | 1 (3%) | 0.99 |  |
| **Other** | **3 (6%)** | **2 (11%)** | **1 (3%)** | **0.27** |  |
| ADL Limitation | 1 (2%) | 1 (6%) | 0 (0%) | 0.35 |  |
| Alopecia | 1 (2%) | 0 (0%) | 1 (3%) | 0.99 |  |
| Flu Like Symptoms | 1 (2%) | 0 (0%) | 1 (3%) | 0.99 |  |
| General disorders | 3 (6%) | 2 (11%) | 1 (3%) | 0.27 |  |
| **Any (not total)** | **34 (65%)** | **9 (50%)** | **25 (74%)** | **0.13** |  |

ADL, activity of daily living; CTCAE, Common Terminology Criteria for Adverse Events; IP, investigational product

^a^ p-values produced by Fisher exact tests

**Supplementary Table 8. Changes in vital signs by treatment group**

|  | **Coefficient (95% CI) (p-value)**^a^ | |
| --- | --- | --- |
| **Contrast**^b^ | **Day 21 vs. Baseline** | **Day 42 vs. Baseline** |
| Temperature |  |  |
| Standard-dose vs. placebo | -0.3 (-1.1, 0.5) (p=0.44) | -0.6 (-1.4, 0.2) (p=0.12) |
| High-dose vs. placebo | -0.6 (-1.4, 0.2) (p=0.13) | -0.4 (-1.1, 0.4) (p=0.38) |
| Pulse |  |  |
| Standard-dose vs. placebo | -1.6 (-11.5, 8.3) (p=0.75) | 2.3 (-7.7, 12.3) (p=0.65) |
| High-dose vs. placebo | -4.9 (-14.8, 5.0) (p=0.33) | -2.3 (-12.2, 7.6) (p=0.65) |
| Systolic Blood Pressure |  |  |
| Standard-dose vs. placebo | 2.4 (-6.7, 11.6) (p=0.6) | 2.8 (-6.5, 12.0) (p=0.56) |
| High-dose vs. placebo | -7.0 (-15.9, 1.9) (p=0.13) | -1.0 (-9.9, 7.9) (p=0.83) |
| Diastolic Blood Pressure |  |  |
| Standard-dose vs. placebo | -0.3 (-7.2, 6.7) (p=0.94) | -1.6 (-8.6, 5.4) (p=0.65) |
| High-dose vs. placebo | -3.2 (-10.0, 3.6) (p=0.36) | -0.8 (-7.6, 6.0) (p=0.82) |

CI, confidence interval

^a^ Between-group differences at follow-up visits estimated using repeated measures linear regression with a random effect for time and represented by marginal means with 95% CIs

^b^ Weight was noted to be significantly higher in the standard-dose group than the placebo group at Day 21 and Day 42, but the recorded values for multiple aberrant weights could not be verified after trial completion. Therefore, we do not report these results in full.
